# The Effects of Gamification on Computerized Cognitive Training: Systematic Review and Meta-Analysis

**DOI:** 10.2196/18644

**Published:** 2020-08-10

**Authors:** Julie F Vermeir, Melanie J White, Daniel Johnson, Geert Crombez, Dimitri M L Van Ryckeghem

**Affiliations:** 1 School of Psychology and Counselling Institute of Health and Biomedical Innovation Queensland University of Technology Brisbane Australia; 2 Science and Engineering Faculty Queensland University of Technology Brisbane Australia; 3 Department of Experimental Clinical and Health Psychology Ghent University Ghent Belgium; 4 Department of Clinical Psychological Science Maastricht University Maastricht Netherlands; 5 Department of Behavioural and Cognitive Sciences University of Luxembourg Esch-sur-Alzette Luxembourg

**Keywords:** gamification, cognition, health, systematic review, meta-analysis

## Abstract

**Background:**

There has been a growing interest in the application of gamification (ie, the use of game elements) to computerized cognitive training. The introduction of targeted gamification features to such tasks may increase motivation and engagement as well as improve intervention effects. However, it is possible that game elements can also have adverse effects on cognitive training (eg, be a distraction), which can outweigh their potential motivational benefits. So far, little is known about the effectiveness of such applications.

**Objective:**

This study aims to conduct a systematic review and meta-analysis to investigate the effect of gamification on process outcomes (eg, motivation) and on changes in the training domain (eg, cognition), as well as to explore the role of potential moderators.

**Methods:**

We searched PsycINFO, Cumulative Index to Nursing and Allied Health Literature, ProQuest Psychology, Web of Science, Scopus, PubMed, Science Direct, Excerpta Medica dataBASE, Institute of Electrical and Electronics Engineers Xplore, Association for Computing Machinery, and a range of gray-area literature databases. The searches included papers published between 2008 and 2018. Meta-analyses were performed using a random-effects model.

**Results:**

The systematic review identified 49 studies, of which 9 randomized controlled trials were included in the meta-analysis. The results of the review indicated that research in this context is still developing and lacks well-controlled empirical studies. Gamification in cognitive training is applied to a large range of age groups and audiences and is mostly delivered at a research site through computers. Rewards and feedback continue to dominate the gamification landscape, whereas social-oriented features (eg, competition) are underused. The meta-analyses showed that gamified training tasks were more motivating/engaging (Hedges g=0.72) and more demanding/difficult (Hedges g=–0.52) than non- or less-gamified tasks, whereas no effects on the training domain were found. Furthermore, no variables moderated the impact of gamified training tasks. However, meta-analytic findings were limited due to a small number of studies.

**Conclusions:**

Overall, this review provides an overview of the existing research in the domain and provides evidence for the effectiveness of gamification in improving motivation/engagement in the context of cognitive training. We discuss the shortcomings in the current literature and provide recommendations for future research.

## Introduction

### Background

Computer-based cognitive training typically involves systematic practice on computerized tasks designed to improve cognitive abilities, such as memory, attention, or processing speed [1]. Although reviews and meta-analyses have demonstrated that such training can be beneficial in enhancing various cognitive functions, interventions are limited by their repetitive and time-consuming character [2,3]. Indeed, most often participants are required to repeat similar training trials continuously over multiple sessions across several weeks. Tasks are perceived as monotonous and boring, which can lead to disengagement and low completion and response rates [4,5]. This is particularly an issue for cognitive research where program adherence is a pertinent problem, with dropout rates often higher than 25% [6,7]. As such, new techniques to help increase task engagement are now being developed [8,9].

There is a growing interest in the application of gamification to computerized cognitive training tasks [10]. Gamification is the use of digital game elements in nonentertainment settings to increase motivation and engagement [10,11]. According to self-determination theory (SDT) [12,13], which is one of the most established theoretical frameworks within gamification research [14], motivation is multidimensional and falls on a continuum from intrinsic motivation through extrinsic motivation to amotivation. Intrinsic motivation (performing an activity for its inherent satisfaction) is a major factor for long-term engagement and long-term behavior change [12,15], whereas extrinsic motivation (performing an activity solely for its outcome) is more useful when short-term engagement and short-term changes are required [13,15]. Gamification aims to combine both types of motivation [16] by using game elements, such as points, badges, game levels, and avatars [10,17].

As the concept of gamification is relatively new [11], empirical evidence regarding the effectiveness of gamified cognitive training tasks is still emerging. The introduction of targeted gamification features to such tasks may increase motivation and engagement as well as improve intervention effects [18,19]. However, there is also the possibility for game elements to have adverse effects on cognitive training (eg, be a distraction), which can outweigh their potential motivational benefits [8]. So far, it is not quite clear how and whether modifying these *traditional* interventions influences motivation, engagement, and training success. Rigorous evaluation of gamified cognitive training tasks is necessary to help guide the design of future tasks, applications, and randomized controlled trials (RCTs).

To our knowledge, there is no meta-analysis of the quantitative body of literature that has specifically examined the effectiveness of gamification applied to cognitive training, nor a review that has examined the quality of evidence for the efficacy of such interventions. However, there has been a systematic review exploring how gamification has been used for cognitive assessment and training purposes, which identified 33 studies [10]. Although the aforementioned review offers valuable insights into how cognitive tasks have been gamified, the quality of evidence provided by the studies was not assessed. Furthermore, the use of gamification in health interventions is a rapidly growing field, especially at a time when there is an increased usage of smart mobile technology, which makes gamified interventions potentially more accessible and appealing [17]. It is therefore timely to conduct an in-depth review of the literature and to provide a meta-analytic synthesis combining all evidence on the effectiveness of gamified cognitive training tasks. Findings from this review may have important implications for technological development, clinical practice, and future research.

### Aims and Objectives

This review was divided into 2 parts. The first part was a comprehensive state-of-the-art review on gamification of cognitive training tasks with the following objectives: (1) determine the types of audiences and age groups that have been investigated; (2) determine the cognitive domains that have been targeted; (3) describe the various forms in which gamification has been applied (eg, type of game elements and theories used); and (4) establish the methodological quality of available studies. The second part is a meta-analysis of the effectiveness of gamification (assessed through RCT studies) applied to cognitive training with the following objectives: (1) assess the impact of gamification on process outcomes (ie, motivation, engagement, flow, immersion, demand, difficulty, and feasibility); (2) assess the impact of gamification on changes in the training domain (ie, cognitive process and clinical outcomes); and (3) explore possible moderators.

## Methods

### Protocol and Registration

This systematic review and meta-analysis were conducted and reported in accordance with the Preferred Reporting Items for Systematic and Meta-Analyses guidelines [20]. Furthermore, the protocol was preregistered in the International Prospective Register of Systematic Reviews (PROSPERO registration number CRD42018082309).

### Definition and Operationalization of Key Constructs

Gamification was defined and operationalized as the use of digital game elements (eg, points, avatars) in nonentertainment settings to increase motivation and engagement [10,11]. This definition allows the differentiation of gamification from serious games [11,21]. Serious games employ full-fledged games within nongame contexts (eg, an interactive world in which players complete challenges designed to improve physical activity), whereas gamification uses elements or individual features of a game embedded in real-world contexts (eg, a mobile health application that uses points and badges to encourage physical activity). In practice, however, the actual distinction between the two can be blurry and highly subjective [21]. As a result, a cautious approach was undertaken in which edge cases were discussed among the authors (JV, DJ, and MW) and resolved by consensus.

Cognitive training was defined and operationalized as training of mental processes involving attention (eg, selective attention), memory (eg, working memory), executive functioning (eg, planning, inhibition, mental flexibility), decision making, processing speed, and perception (including visual, auditory, and tactile perception).

### Search Strategy

Electronic searches were performed between February 14 and 18, 2018. The following electronic databases were included in this review, which were identified as relevant to psychology, health, social science, and information technology: (1) PsycINFO (via EBSCOhost); (2) Cumulative Index to Nursing and Allied Health Literature (CINAHL; via EBSCOhost); (3) ProQuest Psychology; (4) Web of Science; (5) Scopus; (6) PubMed; (7) Science Direct; (8) Excerpta Medica dataBASE (EMBASE); (9) Institute of Electrical and Electronics Engineers (IEEE) Xplore; (10) Association for Computing Machinery (ACM); and (11) a range of gray-area literature databases. A complementary manual search of the reference lists of eligible records and relevant published reviews was also conducted to locate studies not identified in the database searches.

The search terms that we used included terms for gamification in conjunction with terms for cognitive training. Given that gamification was defined and operationalized as the use of digital game elements in nonentertainment settings to increase motivation and engagement [10,11], it was decided to include the term (and variants of the term) *game elements* in the search. This was necessary to capture research that identifies with the literature of gamification but labels it as serious game or games with a purpose rather than gamification. For each database, full and truncated search terms relating to gamification (eg, gamif*, game element*), cognition (eg, cognit*, mental process*), and training (eg, train*, intervention*) were used (see Multimedia Appendix 1 for further details on the search strategy). No limiters were applied at this stage.

### Selection Criteria

For the systematic review, studies were selected if they met the following inclusion criteria:

1. Original empirical research that explicitly refers to gamification, the gamification literature, or the use of game elements. This does not include review papers such as narrative reviews, systematic reviews, or meta-analyses.
2. Peer-reviewed documents (eg, published papers, doctoral theses, study protocols, conference papers).
3. The full text is available in published or unpublished form. This does not include extended abstracts, tutorials, posters, editorials, and letters.
4. A task specifically designed to train or modify cognition as defined earlier. This includes the full range of intervention contexts (eg, health, education, rehabilitation).
5. Cognitive training tasks for which at least one game element (eg, points, avatars) has been added. This does not include studies that reported on persuasive games, serious games, or full-fledged games (eg, video games).
6. Cognitive training tasks are delivered via a digital device (eg, personal computers, laptops, tablets, smartphones, virtual reality headsets).
7. Studies report on at least one outcome related to process (eg, motivation, engagement) or to changes in the training domain (eg, cognition, affect), measured through self-report (eg, questionnaire) and/or objective (eg, dropout rate) measurement methods.
8. The full text is available in English (though research may not necessarily be conducted in English).

For meta-analysis, some additional inclusion criteria were formulated to ensure that studies have a minimal methodological quality. The studies included in the meta-analysis are a subset of the studies included in the systematic review. Additional inclusion criteria for the meta-analysis were as follows:

1. Studies report the effect of (at least) two cognitive training tasks that are similar to each other except for the implementation of gamification features.
2. The study design consisted of an RCT in which 2 groups performed a cognitive training task. Studies were excluded if they only reported a single-group pre-post design.

### Study Selection

Owing to the lack of consensus regarding the definition of gamification, we erred on the side of caution and did not exclude documents based on whether they used terms such as persuasive games, serious games, video games, computer-based, and games with a purpose. Records were selected if they were considered relevant based on our inclusion criteria and on whether at least two authors reached consensus for inclusion.

**Figure 1:**
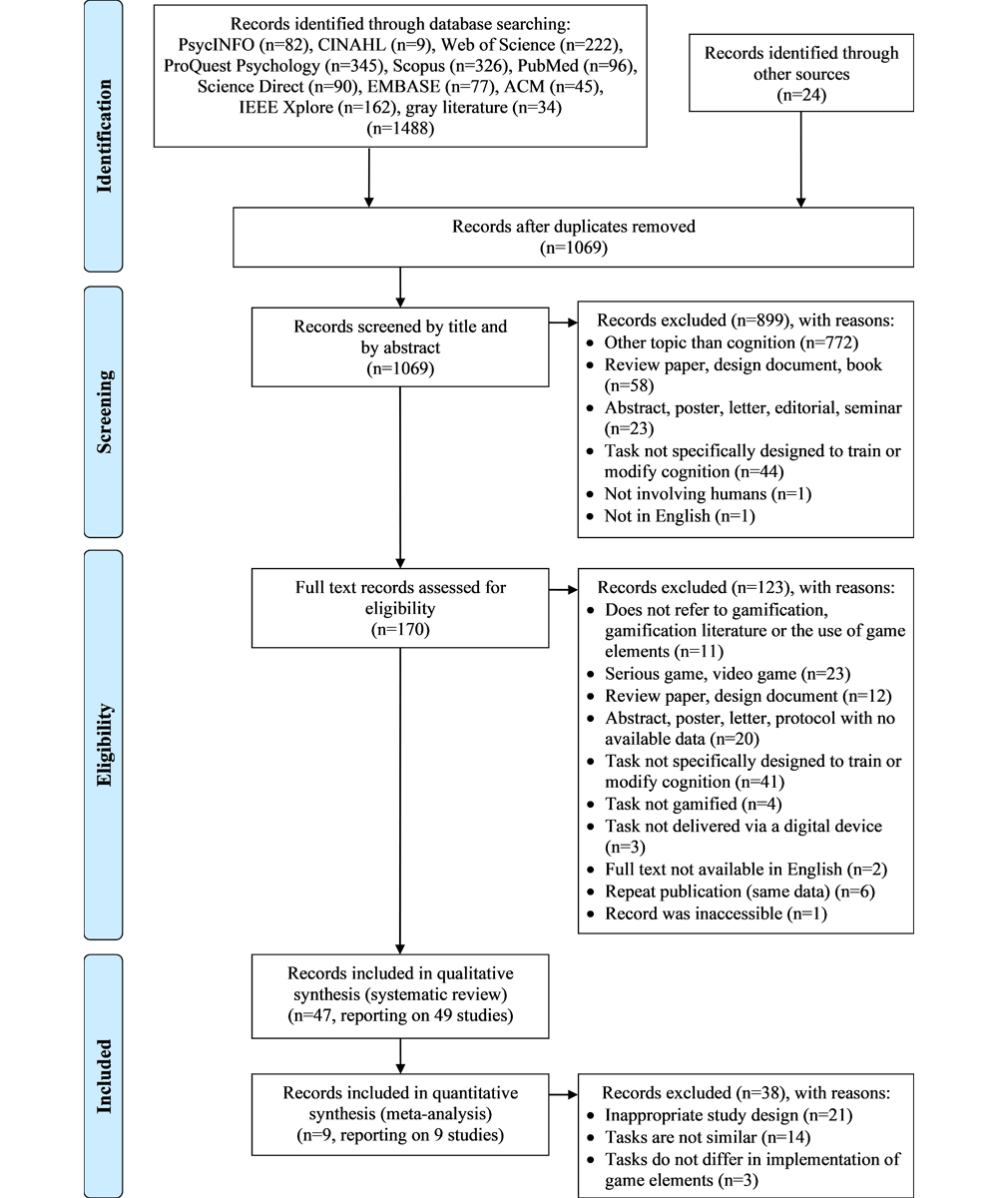
Preferred Reporting Items for Systematic Reviews and Meta-Analyses flowchart of the study selection process.

Figure 1 shows the Preferred Reporting Items for Systematic Reviews and Meta-Analyses flowchart for the selection of included records in the systematic review and meta-analysis. After merging search results across databases (N=1488) using Endnote X8 citation management software, the first author (JV) removed duplicates and screened the remaining records (n=1069) by both title and abstract according to the prespecified eligibility criteria. If it was not possible to determine the eligibility of a record from the title and abstract, the full-text record was obtained. Full-text records (n=170) were retrieved and evaluated against the inclusion criteria (JV). To check the reliability of the process, a second author (MW, DJ, or DV) assessed 20% of the selected full-text records, which resulted in no disagreement. A total of 47 records, reporting on 49 independent studies, were included in the systematic review (Part 1). Of these, 9 records, reporting on 9 independent studies, were suitable for a meta-analysis (Part 2). The list of eligible records was sent to experts in the field of cognitive psychology who were asked to identify further eligible studies, and no relevant records were added. Review authors were not blinded to the authorship, institution, journal, or results.

### Data Extraction Strategy

Data across all included studies were extracted into a prepared Microsoft Access form, which was developed specifically for this review. The data extraction form was piloted on a random sample of studies before being finalized. Two authors (JV and DV) independently extracted and coded the data from the included studies. Any disagreement or ambiguity was resolved by discussion and consensus, and when necessary, a third reviewer (MW, DJ, or GC) was consulted. For the meta-analysis, the corresponding author of each publication was contacted via email for further information when data were missing or unclear. In addition, the authors were invited to comment on the coding and data extraction of their study. When the requested information was not provided, or could not be provided, this was coded as *incomplete data*, and this study was not included in the meta-analyses incorporating this variable. After coding, outcome data for the meta-analysis were extracted and inputted into the Comprehensive Meta-Analysis (CMA) software, version 3.3.070 (Biostat Inc).

### Coding System and Coding Decisions

Every study was coded in terms of study and sample characteristics, intervention characteristics, outcome variables, and methodological quality. For the meta-analysis, additional data were extracted, and several potentially relevant moderating variables were coded.

#### Study and Sample Characteristics

For study characteristics, we provided each study and each outcome within the study with an identification number (eg, study ID1.1, outcome ID1). We then coded the publication channel (published article, doctoral thesis, or conference paper), year of publication, first author’s country of publication, experimental design (between-subject design, single-group design, or case study design), and the number and description of groups. Additional information, such as bibliographic reference and email address of the corresponding author, was also extracted.

For sample characteristics, we coded the overall sample size, the mean age of the sample, proportion of females, and type of participants (eg, trait-anxious adults). For each study included in the meta-analysis, we additionally coded the sample size, mean age of the participants, proportion of females, and sample size used for analyses (ie, n for each outcome) in each condition.

#### Intervention Characteristics

For intervention characteristics, we coded the domain of cognitive training (eg, memory), name of the training task (eg, n-back task), name of the gamified training task (eg, Shots Game), study training length, follow-up(s) after training, number of training sessions, session frequency, average session length (minutes), number of trials per session, type of stimuli used, task delivery location (eg, home), the modality of delivery (eg, tablet), and theory used to apply gamification (eg, SDT). Game elements were coded using a combination of the systems provided by Johnson et al [17], Lumsden et al [10], and Sardi et al [22] (Textbox 1).

**Game elements (adapted from Johnson et al [17], Lumsden et al [10], and Sardi et al [22]) and their descriptions.**

**Avatar**

Visual representation of the user

**Challenge**

Users are required to overcome a challenge by introducing some pressures (eg, time limit, lives) to keep them interested in the gamified system

**Competition**

Users compete against other users. This can be achieved by using, for example, leaderboards

**Difficulty adjustment**

Difficulty is adjusted to the users’ ability either automatically (known as dynamic difficulty adjustment) or manually (eg, users can select the level of difficulty)

**Feedback loops**

Composed of 2 or more steps. Users perform an action, receive gamified/playful feedback about their performance, and with this information can modify their behavior. Feedback loops can be positive (ie, amplifies an action) or negative (ie, reduces an action), and feedback can be provided at different timings (immediate or delayed) and through different delivery methods (eg, visual, auditory)

**Levels**

Visual indicators that inform users about their completed intermediate goals

**Progress (task-related)**

Visual features that inform users about their progression throughout the gamified system (eg, progress bar, a graphical completion chart)

**Rewards**

Indicators such as points (a numerical measure that quantifies users’ participation and performance), badges (visual token of achievements), and other digital rewards (eg, coins, virtual money) are allocated to users for accomplishing certain tasks

**Social interaction**

Users interact with other users

**Sound effects**

Noises used to enhance the experience (eg, music background) or to reinforce a specific action (eg, buzz sound for incorrect response)

**Story/theme**

Graphics or text linked together within the gamified system to enhance the experience or to give meaning to tasks (also known as meaningful stories)

#### Outcome Variables

For the systematic review, study outcome(s) and measurement instruments used for assessing these outcomes were extracted for each study. For the meta-analysis, some additional data extraction and coding decisions were made. In particular, we decided to take a more refined approach to the categorization of outcomes stated in the study protocol (CRD42018082309). Process-related outcomes were categorized as motivation/engagement (including interest and enjoyment), flow/immersion, demand/difficulty, and feasibility (including ease of use and task clarity). Training domain–related outcomes were categorized as a cognitive process (eg, alcohol attentional bias, working memory) and clinical outcomes (eg, alcohol use, psychiatric symptoms). This final categorization was based on consensus discussions among the authors of this paper, who have research expertise in the field of gamification and cognitive psychology. To calculate effect sizes for each of these outcomes, we opted to use only the posttraining data (ie, means, standard deviations) rather than change scores (ie, the difference between pre- and posttraining data). This approach reduces the risk of selective reporting [23,24]. Next, categorical process variables having more than two categories [25] were recoded into dummy variables, with 0 as the less desirable outcome for gamification and 1 as the more desirable outcome for gamification. Finally, when a study reported results of subgroups instead of the overall results for a group of interest, we calculated the combined effect size using the available formula in the Cochrane Handbook [23] and imputed the pooled mean and standard deviation in the meta-analysis.

#### Quality Assessment of Included Studies

For the systematic review (Part 1), we assessed the methodological quality of each study using a quality assessment tool specifically designed for this review. Judgments of external validity were based on the description of the setting and/or location of the study and the description of the gamified task, both coded either as *adequate* or *not adequate*. The first item was coded as *adequate* if the study provided descriptions of the following features: (1) setting where participants were recruited (eg, general population); (2) type of participants (eg, undergraduates of university); and (3) location where the study took place (eg, laboratory). The second item was coded as *adequate* if the study provided at least a description of the following features: (1) the number of trials or duration of the task; (2) type of response required during the task (eg, indicate whether the arrow is pointing to the left or the right); and (3) game elements used (described using a picture or text). The criteria for internal validity were related to the description of blinding of participants (blinding of participants, no blinding of participants, or unclear) and personnel (blinding of personnel, no blinding of personnel, or unclear), and the inclusion of a comparison control condition (yes or no). Judgments were made by 2 authors (JV and DV) independently, and a consensus was reached for existing discrepancies through discussion.

For the meta-analysis (Part 2), we used the Cochrane Collaboration risk of bias tool as described in the Cochrane Handbook for Systematic Reviews of Interventions [26]. The tool covers 5 biases: selection bias (random sequence generation and allocation concealment), performance bias (blinding of participants and personnel), detection bias for each outcome separately (blinding of outcome assessment), attrition bias (incomplete outcome data), and selective outcome reporting bias. The risk of bias for each domain was then judged as either *low risk*, *high risk*, or *unclear risk*. Judgments were made by 2 authors (JV and DV) independently, using the criteria for assessing the potential risk of bias [26]. The consensus was reached for all discrepancies through discussion.

#### Coding of Moderators (Meta-Analysis)

The present meta-analysis examined several samples, intervention, and study characteristics as potential moderators of the effects of gamification on outcomes related to process and changes in the training domain. Specifically, because research has shown that there may be age differences in perceived benefits from the use of gamification [27], we investigated age groups (children and adolescents: mean age <18 years or adults: mean age ≥18 years) as a potential moderator. In addition, we investigated cognitive domain targeted and population targeted (low-risk group or high-risk group) as potential moderators. Studies coded with participants in the high-risk group include at-risk participants such as the elderly, people with specific health issues (eg, patients with gliomas), or people selected because of greater health risks (eg, heavily drinking students), whereas studies coded with participants in the low-risk group included participants who were not preselected due to health risks (eg, university students, primary school children). With regard to study characteristics, we examined the number of training sessions. The question can be raised whether one session is enough to see changes in the training domain targeted. Gamification can add complexity to the training environment, and game elements may initially distract participants from the core task [8]. Thus, it is possible that training effects occur only after multiple training sessions in which participants become familiar with the gamified environment. In contrast, motivation and excitement may be high in the first few sessions but decline over time when novelty decreases [22].

Finally, we explored gamification elements (ie, number and type of game elements) as potential moderators. Similar to Koivisto and Hamari [28], game elements were grouped based on their type into the following: achievement and progression-oriented (rewards, challenge, game level, progress, difficulty adjustment, feedback loops); social-oriented (competition, social interaction); or immersion-oriented (avatar, story/theme, sound effects). This decision was made due to studies using different combinations of game elements, making the analysis of individual game elements unfeasible.

### Meta-Analytic Procedures

To investigate the effectiveness of gamification applied to cognitive training tasks, 2 separate sets of meta-analyses were conducted. The first set assessed the impact of gamification on process-related outcomes (ie, motivation, engagement, flow, immersion, demand, difficulty, and feasibility) and variables moderating this relationship. The second set of meta-analyses explored the effects of gamification on changes in the training domain (ie, cognitive process and clinical outcomes) and variables moderating this relationship.

A random-effects model was chosen to combine effect sizes from different studies. We used Hedges g*,* which corrects for small sample sizes, to calculate effect sizes for outcome data based on posttraining means and standard deviations [29]. A negative or positive Hedges g (significant at *P*<.05) indicated that the gamified condition performed more poorly or better than the control condition (ie, a non- or less- gamified version), respectively. The degree of heterogeneity in effect sizes (significant at *P*<.05) was assessed using the Cochran Q test [30].

The sources of heterogeneity were explored by conducting subgroup and meta-regression analyses. For moderator analyses with categorical coded variables (eg, age group), we used a mixed-effects model and opted to pool within-group estimates of Tau-squared due to the limited number of studies per condition. Heterogeneity within each group (Q_w_) and heterogeneity between groups (Q_b_) were also evaluated. A significant Q_b_ (*P*<.05) indicates a significant difference in the magnitude of the effect sizes between categories of the moderator variable. If there were less than three studies within a subgroup, moderator analyses were not conducted. To maintain the independence of data, whenever necessary, effect sizes were averaged across different outcomes. For the coded continuous variables (eg, number of training sessions), we performed meta-regressions using the method-of-moments procedure with the Knapp-Hartung correction, where the slope (β) and its *P* value indicated the importance of this moderator in understanding linear changes in effect sizes [31].

Publication bias was assessed by visual inspection of funnel plots and the Egger regression test, with asymmetric funnel plots and the significant Egger test (*P*<.05) indicating publication bias [32]. When the funnel plot inspection or Egger test suggested the presence of publication bias, the trim-and-fill procedure by Duval and Tweedie [33] was applied. This procedure provides an adjusted estimate of effect size after accounting for publication bias. Publication bias analyses were conducted if at least three studies were available in the overall meta-analyses. Finally, a leave-one-out sensitivity analysis was performed by iteratively removing one study at a time to confirm that our findings were not driven by any single study. All analyses were performed using the CMA software. The magnitude of the effect sizes was categorized as small (≥0.20 to <0.50), moderate (≥0.50 to <0.80), or large (≥0.80) [34].

## Results

### Systematic Review of the Gamification of Cognitive Training (Part 1)

#### Summary of Included Studies

The key characteristics of the 49 studies included in the systematic review are summarized in Multimedia Appendix 2. A total of 78% (n=38/49) of articles were published in academic journals, 18% (n=9/49) were conference papers, and 4% (n=2/49) were doctoral theses. Studies were published between 2008 and 2017, with the majority (n=46/49, 94%) published after 2010, indicating that research on gamified cognitive training tasks is a young but rapidly growing field of research. Most of the studies (n=32/49, 65%) were undertaken in European countries, 25% (n=12/49) in the United States, 4% (n=2/49) in Australia, 4% (n=2/49) in Asian countries, and 2% (n=1/49) in Canada.

The included studies involved a total of 4003 participants. Sample sizes ranged from 3 [35,36] to 794 [37] participants. Notably, most studies (n=47/49, 96%) had fewer than 200 participants in total (often split over different conditions). When reported (n=44/49, 90%), the mean age of the participants varied from 8.98 [38] to 82.70 years [39], showing that gamification has been applied to a large range of age groups. The majority of the studied samples (n=43/49, 88%) included both males and females, in which the proportion of females varied from 9% [40] to 93% [39]. Two studies focused on male-only samples [35,41], 1 study only included female participants [42], and 3 studies did not specify the gender ratio [43-45]. Over three-fourth (n=38/49, 78%) of the studies were conducted with adults, whereas the remaining one-fourth involved children and adolescents. Of the 49 studies, 15 (31%) were delivered to low-risk samples (eg, university students, schoolchildren), 33 (67%) to high-risk groups (eg, elderly drivers, children with autism spectrum disorder), and 1 study involved both populations [46].

A range of study designs were represented across the 49 studies, with the majority (n=40/49, 82%) using a between-group (pre-post or post only) design with 2, 3, 4, 6, or 7 groups. Other study designs consisted of single-group designs (n=7/49, 14%) and case study designs (n=2/49, 4%). A total of 31 (63%) studies conducted single-domain cognitive training, with attention (n=11/49, 22%) and working memory (n=9/49, 18%) being most often targeted. The remaining studies trained multiple domains of cognition simultaneously. With respect to the reported outcomes, research on gamified cognitive training tasks is diverse. Some studies explored the effects of gamification on process-related outcomes (eg, motivation, engagement, feasibility), whereas others investigated changes in the training domain (eg, cognition, anxiety, attention-deficit/hyperactivity disorder [ADHD] symptoms); others reported a mixture of outcomes. There was also a broad variety of outcome measures used across the studies (eg, questionnaires, semistructured interviews, video-recorded observations). The final coding scheme for outcome measures is available from the authors upon request.

Overall, interventions ranged in length from one session (range 10-80 min) to multiple sessions (maximum 59 sessions; range 5-72 min), lasting up to 18 months [37]. All studies evaluated outcomes at the end of the training, but only about one-third (n=14/49, 29%) reported additional follow-up assessments, which ranged from 1 week ([47], study 1) to 5 years [48]. When the study location was provided (n=42/49, 86%), interventions were delivered at a research site (eg, laboratory, hospital, school; n=19/49, 39%), at participants’ homes (n=10/49, 20%), web-based (n=5/49, 10%), at a location of choice (n=1/49, 2%), or a mixture thereof (eg, laboratory and/or home; n=7/49, 14%). The chief modalities employed for delivering gamified cognitive training were the following: (1) computers (ie, desktop, laptop, and notebook; n=26/49, 53%); (2) tablets (n=13/49, 27%); (3) smartphones (n=2/49, 4%); (4) and iPod touch (n=2/49, 4%). Several studies (n=5/49, 10%) used a combination of devices (eg, computer or tablet), and one study did not provide information on the mode of delivery [49].

The 49 studies included in this systematic review employed a range of game elements embedded in a variety of ways. All studies incorporated a combination of game elements into each intervention (range 2-9) and the mode count of gamification features was 5 (Multimedia Appendix 2). The most frequently used game elements were rewards (eg, points, badges), followed by feedback loops, story/theme, and difficulty adjustment (Table 1). Most of these game elements can be categorized as achievement and progression-oriented game features, which form the most common category of game elements used in cognitive training. Interestingly, only around one-third (n=17/49, 35%) of the studies draw upon existing theories and principles for designing gamified interventions; specifically, SDT [12,13] (n=3/49, 6%), flow theory [50] (n=2/49, 4%), the framework by Kiili and Perttula [51] (n=2/49, 4%), operant conditioning (n=1/49, 2%), principles by Gee [52] (n=1/49, 2%), or a combination of several theories and principles (n=8/49, 16%).

**Table 1:** Type of game elements used in the selected studies.

Game elements		Count, n
**Achievement and progression-oriented**		
	Rewards	40
	Feedback loops	39
	Difficulty adjustment (dynamic and/or manual)	34
	Game level	26
	Progress	25
	Challenge	24
**Social-oriented**		
	Competition	3
	Social interaction	2
**Immersion-oriented**		
	Story/theme	38
	Sound effects	19
	Avatar	16

#### Quality of Included Studies

The ratings of methodological quality for studies included in the systematic review are provided in Multimedia Appendix 2. Overall, all studies (with the exception of that of Mohammed et al [53]) had at least one of the applicable criteria not fulfilled. Regarding external validity, most studies adequately described the setting and/or location of the study (n=40/49, 82%) and the gamified task (n=43/49, 88%). However, for the internal validity criteria, most studies had lower quality assessment ratings due to inadequate or unclear blinding of participants (n=38/49, 78%) and personnel (n=39/49, 80%), and the absence of a control (ie, a non- or less- gamified version) comparison condition (n=40/49, 82%).

### Meta-Analysis of the Effectiveness of Gamified Cognitive Training (Part 2)

#### Effects of Gamification on Process-Related Outcomes

##### Motivation/Engagement

The meta-analysis on the effect of gamification on motivation/engagement-related outcomes contained 8 independent studies, involving a total of 514 participants, with 295 participants (M_age_=18.88; M_%females_=48) in the gamified groups and 219 participants (M_age_=19.23_;_ M_%females_=56) in the comparison groups (ie, non- or less-gamified version).

**Figure 2:**
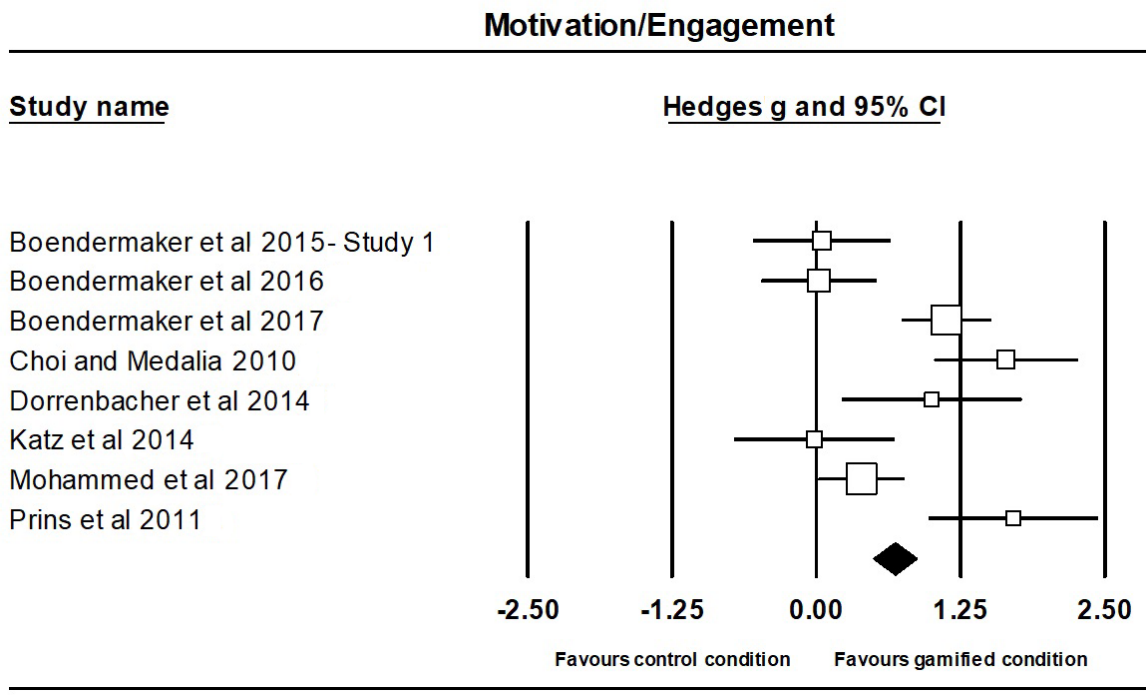
Forest plot of overall effect sizes comparing gamified condition and control condition (ie, non- or less-gamified version) on motivation/engagement for individual studies in alphabetical order.

The effect of gamification on motivation/engagement was moderate, positive, and significant (Hedges g=0.72; 95% CI 0.26 to 1.19; *P*=.002), indicating that gamified tasks were more motivating/engaging than non- or less-gamified tasks (Figure 2). There was significant heterogeneity between studies (Q_7_=39.73; *P*<.001), warranting the relevance of moderator analyses (see section Subgroup and Meta-Regression Analyses). A sensitivity analysis revealed that the results were not significantly altered by the removal of any one study, indicating the reliability of the findings (Multimedia Appendix 3). A visual inspection of the funnel plot (Multimedia Appendix 3) and Egger test (*t*_6_=0.37; *P*=.72) showed that there was no evidence of publication bias.

##### Flow/Immersion

The meta-analysis on the effect of gamification on flow/immersion-related outcomes contained 2 independent studies, involving a total of 79 participants, with 47 participants (M_age_=22.85; M_%females_=49) in the gamified groups and 32 participants (M_age_=23.69; M_%females_=72%) in the comparison groups (ie, non- or less-gamified version).

**Figure 3:**
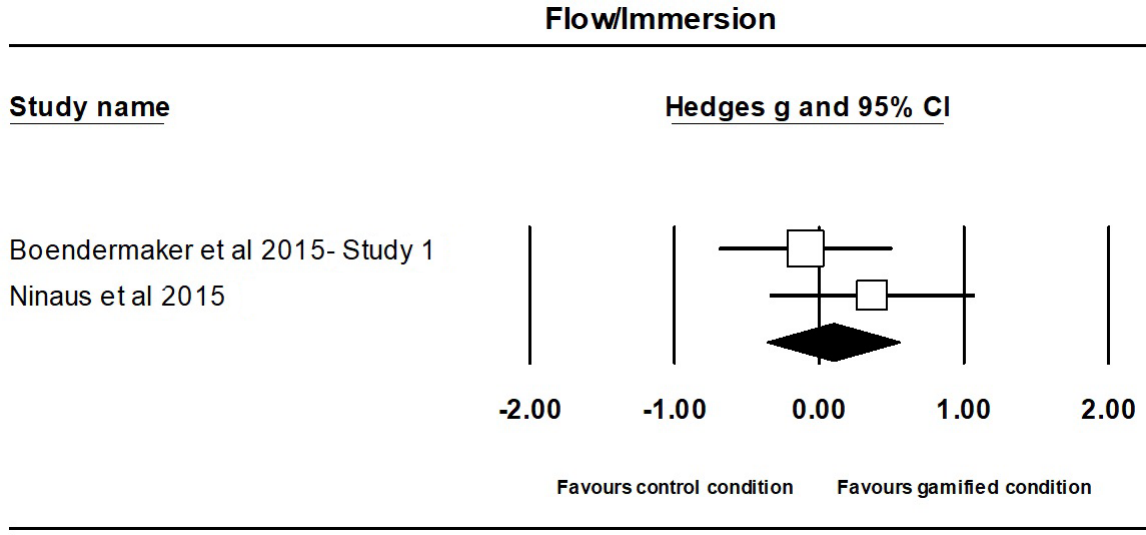
Forest plot of overall effect sizes comparing gamified condition and control condition (ie, non- or less-gamified version) on flow/immersion for individual studies in alphabetical order.

The effect of gamification on flow/immersion was not significant (Hedges g=0.10; 95% CI 0.36 to 0.55; *P*=.68; Figure 3), with no evidence of significant heterogeneity (Q_1_=0.95; *P*=.33). There have been too few studies to allow the assessment of publication bias and to perform a sensitivity analysis.

##### Demand/Difficulty

The meta-analysis on the effect of gamification on demand/difficulty-related outcomes contained 3 independent studies, involving a total of 136 participants, with 86 participants (M_age_=15.24; M_%females_=35) in the gamified groups and 50 participants (M_age_=13.60; M_%females_=26) in the comparison groups (ie, non- or less-gamified version).

**Figure 4:**
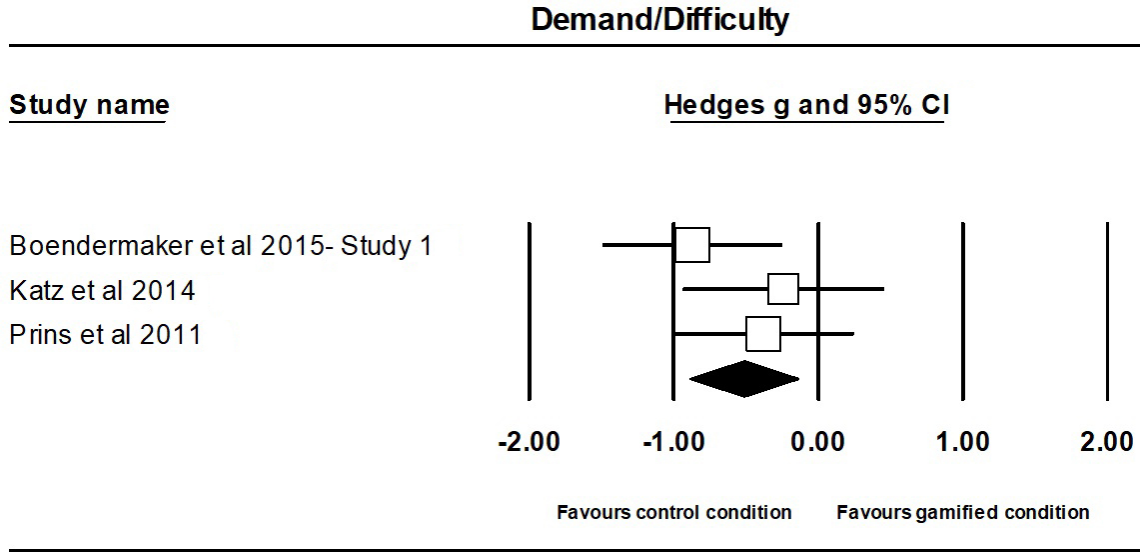
Forest plot of overall effect sizes comparing gamified condition and control condition (ie, non- or less-gamified version) on demand/difficulty for individual studies in alphabetical order.

The effect of gamification on task demand/difficulty was moderate, negative, and significant (Hedges g=0.52; 95% CI 0.89 to 0.14; *P*=.007), indicating that gamified tasks were more demanding/difficult than non- or less-gamified tasks (Figure 4). There was no evidence of significant heterogeneity between studies (Q_2_=2.03; *P*=.36). A sensitivity analysis showed significant changes in the results, possibly because of the low statistical power of the analysis of 2 studies only (Multimedia Appendix 3). A visual inspection of the funnel plot (Multimedia Appendix 3) and Egger test (*t*_1_=0.91; *P*=.53) showed that there was no evidence of publication bias.

##### Feasibility-Related Outcomes

The effect of gamification on feasibility was only reported in 1 study ([47], study 1). The results of this study indicated that gamified tasks (n=34; M_age_=22.64; M_%females_=44) were less easy to use or less clear than the control task (n=15; M_age_=23.20; M_%females_=47); however, this difference was not significant (Hedges g =−0.36; 95% CI −0.97 to 0.26; *P*=.25). There have been too few studies to perform any further analyses.

#### Effects of Gamification on Training Domain–Related Outcomes

##### Cognitive Process

The meta-analysis on the effect of gamification on cognitive process outcomes contained 9 independent studies, involving a total of 530 participants, with 301 participants (M_age_=19.10; M_%females_=49) in the gamified groups and 229 participants (M_age_=9.73; M_%females_=59) in the comparison groups (ie, non- or less-gamified version).

**Figure 5:**
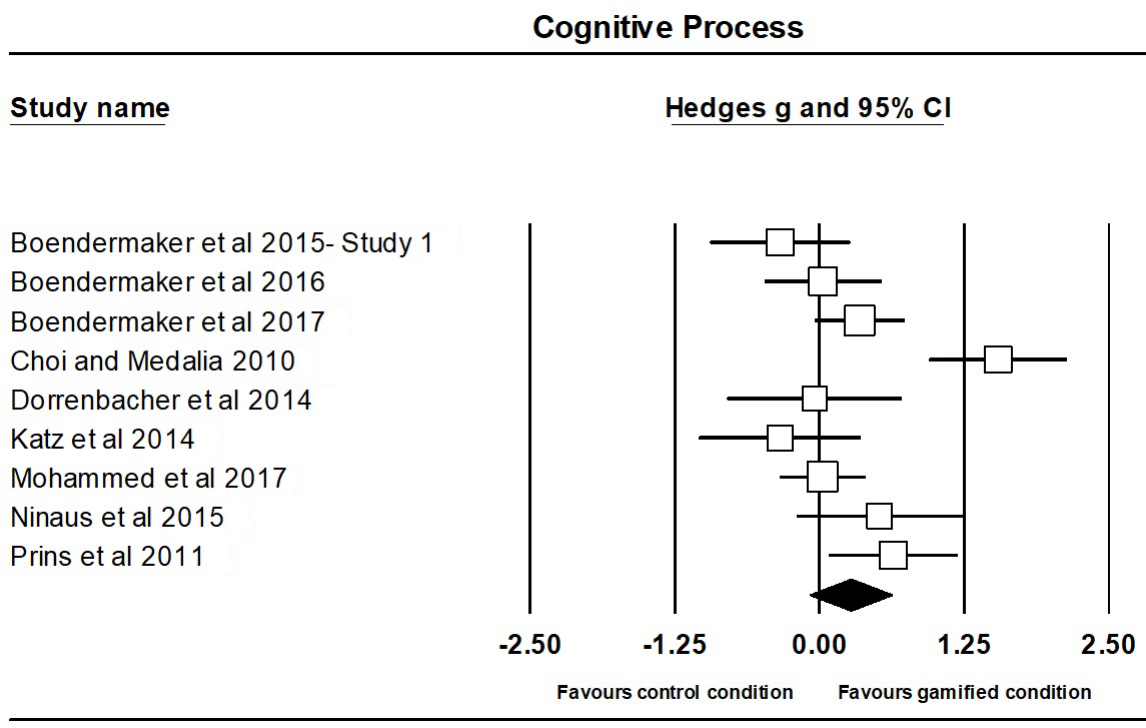
Forest plot of overall effect sizes comparing gamified condition and control condition (ie, non- or less-gamified version) on the cognitive process for individual studies in alphabetical order.

The effect of gamification on cognitive process outcomes was small and positive, but not significant (Hedges g=0.27; 95% CI 0.08 to 0.62; *P*=.14; Figure 5). There was significant heterogeneity between studies (Q_8_=29.91; *P*<.001), warranting the relevance of moderator analyses (see section Subgroup and Meta-Regression Analyses). A sensitivity analysis revealed that the results were not significantly altered by the removal of any one study (Multimedia Appendix 3). Visual inspection of the funnel plot (Multimedia Appendix 3) and Egger test (*t*_7_=0.15; *P*=.88) showed that there was no evidence of publication bias.

##### Clinical Outcomes

The meta-analysis on the effect of gamification on clinical outcomes contained 4 independent studies, involving a total of 276 participants, with 158 participants (M_age_=21.88; M_%females_=2) in the gamified groups and 119 participants (M_age_=22.94; M_%females_=65) in the comparison groups (ie, non- or less-gamified version).

**Figure 6:**
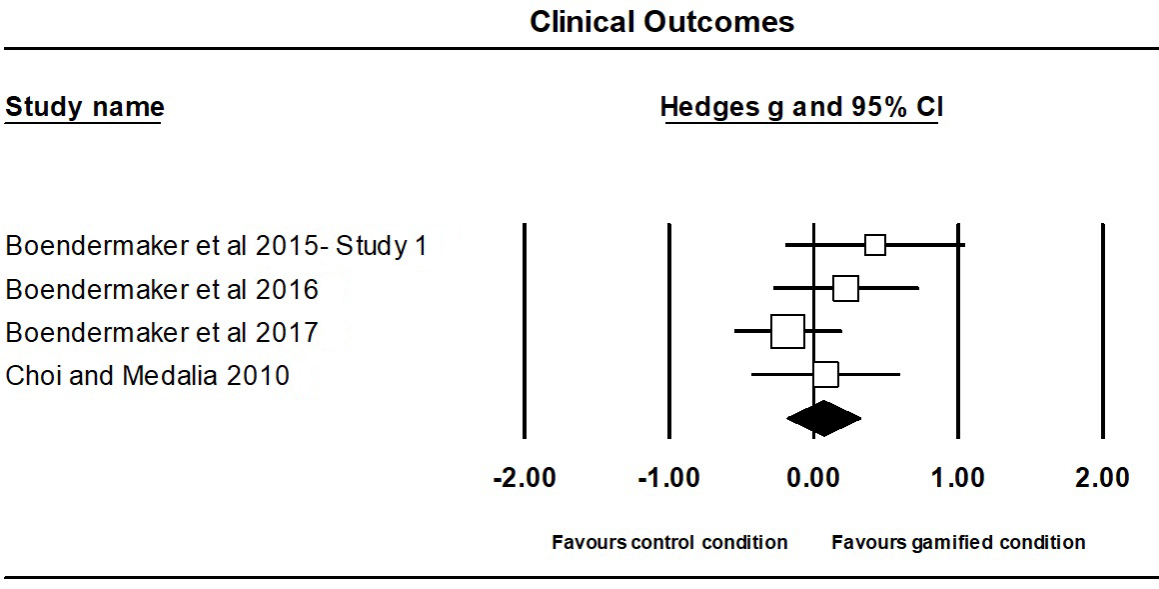
Forest plot of overall effect sizes comparing gamified condition and control condition (ie, non- or less-gamified version) on clinical outcomes for individual studies in alphabetical order.

The effect of gamification on clinical outcomes was not significant (Hedges g=0.07; 95% CI 0.19 to 0.32; *P*=.61; Figure 6), with no evidence of significant heterogeneity (Q_3_=3.32; *P*=.35). A sensitivity analysis revealed that the results were not significantly altered by the removal of any 1 study (Multimedia Appendix 3). A visual inspection of the funnel plot (Multimedia Appendix 3) and Egger test (*t*_2_=5.59; *P*=.03) showed that there was evidence of publication bias. The analyses undertaken using the trim-and-fill approach by Duval and Tweedie [33] did not change the overall effect (Hedges g=0.08; 95% CI 0.36 to 0.20; 2 studies were trimmed). Follow-up effect sizes for clinical outcomes were not calculated because only 1 study reported follow-up [54].

#### Subgroup and Meta-Regression Analyses

To test possible explanations of observed heterogeneity for motivation/engagement and cognitive process outcomes, several subgroup analyses and meta-regressions were conducted.

##### Age Group

Subgroup analyses were performed to examine whether effect sizes varied according to age category (children and adolescents vs adults). There was no significant difference in effect sizes between studies targeting children and adolescents and studies targeting adults for motivation/engagement outcomes (Q_1_=0.94; *P*=.33) and cognitive process outcomes (Q_1_=0.16; *P*=.69; Tables 2 and 3).

**Table 2:** Results of the subgroup analyses for motivation/engagement outcomes.

Moderator		n^a^	k^b^	g^c^	95% CI	*P* value	Q_w_^d^	*P* value	Q_b_^e^	*P* value
**Cognitive domain**									—^f^	—
	Arithmetic ability	57	1	1.64	0.13 to 3.16	.03	—	—		
	Attention	61	1	0.02	-1.45 to 1.49	.98	—	—		
	Executive control	27	1	1.00	-0.59 to 2.59	.22	—	—		
	Inhibition	169	2	0.62	-0.43 to 1.65	.25	8.84	.003		
	Working memory	200	3	0.67	-0.20 to 1.54	.13	12.74	.002		
**Age group**									0.94	.33
	Adults	282	4	0.51	-0.12 to 1.13	.11	18.86	<.001		
	Children and adolescents	232	4	0.96	0.30 to 1.61	.004	12.19	.007		
**Population type**									0.14	.71
	Low risk	298	4	0.63	-0.08 to 1.34	.08	11.99	.007		
	High risk	216	4	0.82	0.10 to 1.54	.03	27.74	<.001		
**Game elements type**									—	—
	Achievement, progression, and immersion	408	6	0.69	0.12 to 1.26	.02	26.12	<.001		
	Achievement, progression, immersion, and social	106	2	0.84	-0.15 to 1.83	.10	13.14	<.001		

^a^n: combined sample size.

^b^k*:* number of studies.

^c^g: Hedges g.

^d^Q_w_*_:_* heterogeneity statistics within each group.

^e^Q_b:_ heterogeneity statistics between groups.

^f^Not available due to insufficient observations.

**Table 3:** Results of the subgroup analyses for cognitive process outcomes.

Moderator		*n* ^a^	*k* ^b^	*g* ^c^	95% CI	*P* value	*Q*_*w*_^d^	*P* value	*Q*_b_^e^	*P* value
**Cognitive domain**									—^f^	—
	Arithmetic ability	57	1	1.54	0.66 to 2.43	.001	—	—		
	Attention	61	1	0.03	–0.80 to 0.86	.94	—	—		
	Executive control	26	1	–0.04	–1.04 to 0.96	.93	—	—		
	Inhibition	155	2	0.06	–0.52 to 0.64	.85	3.53	.06		
	Working memory	231	4	0.21	–0.23 to 0.64	.35	6.28	.10		
**Age group**									0.16	.69
	Adults	310	5	0.34	–0.17 to 0.85	.20	24.46	<.001		
	Children and adolescents	220	4	0.18	–0.40 to 0.76	.54	5.44	.14		
**Population type**									0.89	.35
	Low risk	314	5	0.11	–0.38 to 0.60	.66	4.60	.33		
	High risk	216	4	0.46	–0.08 to 1.00	.10	22.58	< .001		
**Game elements type**									—	—
	Achievement, progression, and immersion	425	7	0.18	–0.22 to 0.58	.39	7.66	.26		
	Achievement, progression, immersion, and social	105	2	0.61	–0.16 to 1.37	.12	18.94	< .001		

^a^*n*: combined sample size.

^b^*k:* number of studies.

^c^*g*: Hedges *g*.

^d^Q_w:_ heterogeneity statistics within each group.

^e^Q_b:_ heterogeneity statistics between groups.

^f^Not available due to insufficient observations.

##### Population Type

Subgroup analyses were performed to examine whether effect sizes varied according to the population type (low-risk vs high-risk groups). There was no significant difference in effect sizes between training tasks targeting low-risk samples (eg, university students) and tasks targeting high-risk groups (eg, elderly drivers) for motivation/engagement outcomes (Q_1_=0.14; *P*=.71) and cognitive process outcomes (Q_1_=0.89; *P*=.35; Tables 2 and 3).

##### Cognitive Domain

Subgroup analyses according to the cognitive domain targeted were not possible because there were fewer than 3 studies within some subgroups for both motivation/engagement and cognitive process outcomes (Tables 2 and 3).

##### Game Elements Type

Subgroup analyses according to the type of game elements used were not possible because there were fewer than 3 studies within a subgroup for both motivation/engagement and cognitive process outcomes (Tables 2 and 3). It is noteworthy that, based upon categorization, we were unable to investigate the impact of a single category (eg, achievement and progression features) because studies always incorporated a combination of categories into their tasks. In particular, studies reported only outcomes from the following combined categories: (1) achievement, progression, and immersion-oriented game features and (2) achievement, progression, immersion, and social-oriented game features (Tables 2 and 3).

##### Number of Game Elements

Meta-regression analyses were performed to examine whether the effects of gamification on outcomes of interest varied according to the number of game features used (independent of the game element type). There was no significant relationship between the number of game elements used and effect sizes (Hedge g) for motivation/engagement outcomes (point estimate of slope=0.26; 95% CI 0.57 to 0.06; *P*=.09; Multimedia Appendix 3) and cognitive process outcomes (point estimate of slope=0.21; 95% CI 0.46 to 0.04; *P*=.08; Multimedia Appendix 3).

##### Number of Training Sessions

Meta-regression analyses were performed to examine whether the effects of gamification on outcomes of interest varied according to the number of training sessions performed. There was no significant relationship between the number of training sessions and effect sizes for motivation/engagement outcomes (point estimate of slope=0.0002; 95% CI 0.12 to 0.12; *P*=.99; Multimedia Appendix 3) and cognitive process outcomes (point estimate of slope=0.01; 95% CI 0.07 to 0.09; *P*=.78; Multimedia Appendix 3). Of note is the low variability in the number of training sessions.

#### Quality of Included Studies

The risk of bias judgments for each included study is summarized in Table 4. Overall, the quality of the included RCTs was not optimal. The most common risks of bias were inadequate blinding of participants, personnel, and outcome assessors. The results did, however, reveal a low risk of bias for random sequence generation and selective reporting. It is noteworthy that for handling incomplete outcome data, all studies (with the exception of that of Mohammed et al [53]) did not provide the information necessary for assessing whether the criteria were met.

**Table 4:** Risk of bias assessment of the included studies.

Study^a^	Random sequence generation (selection bias)	Allocation concealment (selection bias)	Blinding of participants and personnel (performance bias)	Blinding of outcome assessment (detection bias)	Incomplete outcome data (attrition bias)	Selective reporting (reporting bias)
Boendermaker et al [47] study 1	Low	Low	High	High	Unclear	Low
Boendermaker et al [54]	Low	Low	High	High	Unclear	Low
Boendermaker et al [55]	Low	Low	High	High	Unclear	Low
Choi and Medalia [56]	Low	High	High	High	Unclear	Low
Dorrenbacher et al [57]	High	High	High	High	Unclear	Unclear
Katz et al [8]	High	High	Unclear	Unclear	Unclear	Unclear
Mohammed et al [53]	Low	High	Low	Low	Low	Low
Ninaus et al [18]	Low	Unclear	High	Low	Unclear	High
Prins et al [25]	Unclear	Unclear	High	Unclear	Unclear	Unclear

^a^Risk of bias assessment using the Cochrane risk of bias tool.

## Discussion

### Principal Findings

The aims of this study were to provide a state-of-the-art review of the gamification of cognitive training tasks and to evaluate its effectiveness. The systematic review identified 49 independent studies, with the majority published after 2010 (n=46/49, 94%) and conducted in European countries (n=32/49, 65%). The most comparable study is a systematic review by Lumsden et al [10] of gamified cognitive assessment and training tasks, which identified 33 studies published between 2007 and 2015. This indicates that the gamification of cognitive training is a rapidly evolving area of research. However, little is known about the effectiveness of such interventions. Only 9 (18%) of the 49 studies allowed rigorous testing of intervention effectiveness and were included in the meta-analysis, showing that the field is still developing and lacks well-controlled empirical studies. Overall, results from the meta-analysis of RCTs showed that gamified training tasks were more motivating/engaging and more demanding/difficult than non- or less-gamified tasks. No other significant effects were found, and no moderator variables were identified. However, the findings were limited by the small number of studies. The following sections discuss the key results obtained from the systematic review and meta-analysis and provide directions for future research.

#### Effectiveness of Gamification Applied to Cognitive Training

Gamification is commonly framed as a technique for increasing motivation and engagement in a given task [28]. The results from the meta-analysis suggest significant and moderate positive effects of gamification on motivation/engagement outcomes (N=8 studies; Hedges g=0.72; *P*=.002). This provides the first evidence that the application of gamification in cognitive training can positively influence motivation/engagement and confirms the findings of an earlier review by Lumsden et al [10], which also found evidence for the potential of gamification to enhance motivation and engagement in cognitive tasks. Our analysis, however, also indicates that gamified tasks are perceived as significantly more demanding/difficult. This is relatively unsurprising given that implementing game features adds a layer of complexity to a task [58]. However, the result is based on the synthesis of only 3 studies, and it remains unclear whether this affects training domain outcomes. Therefore, further research is warranted in this area.

Interestingly, we found no significant effect of gamification on cognitive outcomes (N=9 studies; Hedges g=0.27; *P*=.14). Thus, overall, gamification does not seem to have enhancing or deleterious effects on cognitive performance. Previous systematic reviews in the health [17] and cognitive [10] fields have reported that the evidence for the impact of gamification on cognitive outcomes is mixed. Some critical views on gamification interpret such findings as an illustration that it does not work [59], whereas others argue that these mixed results are due to confounders such as poor design [60,61] or failure to apply gamification in an appropriately considered or meaningful way [15]. In fact, the implementation of gamification has been cataloged within the group of complex interventions [60]. Complex interventions are activities that comprise multiple interacting components (eg, intensity, setting) that, when applied to the target population, result in variable outcomes [62]. Therefore, it is recommended that researchers developing and evaluating gamified health-related interventions consider using a framework, such as the Medical Research Council framework for complex interventions [62,63], to ensure that the implementation of game features has been well-thought through, and therefore, be more likely to produce the desired outcomes.

For both motivation/engagement and cognitive process outcomes, we found considerable heterogeneity in the results, which we were unable to explain by a systematic consideration of the age group, target population, number of game elements, and number of training sessions. Owing to insufficient studies, we were unable to examine the types of game elements or cognitive domains as potential moderators of outcomes. The results of these moderator analyses are further discussed in the following sections.

#### Audiences and Age Groups Investigated

In the systematic review, studies generally had small sample sizes, ranging widely from 3 [35,36] to 794 [37] participants, with nearly half (n=22/49, 45%) of the studies having fewer than 50 participants in total. In line with previous reviews [10,17], we found that gamification was applied to a large range of age groups, from children ([64], study 2) through adolescents [55] to adults [65]. Adults were the main target audience (n=38/49, 78%); however, we also found that more research findings have started to attach importance to the use of gamification for children and adolescents since 2011. With regard to the type of population investigated, most (n=33/49, 67%) of the interventions were delivered to high-risk groups, such as older adults [41] and individuals with specific health issues such as ADHD [66] or moderate levels of trait anxiety [67]. The remaining studies targeted low-risk samples such as university students [18] and school children [38].

In the meta-analysis, the findings that neither age nor population type had any moderating effects on motivation/engagement and cognitive process outcomes indicate that gamification may be equally applicable to a broad range of individuals. However, this does not imply that users should be treated as a monolithic group nor that researchers should adopt a *one-size-fits-all* design approach (ie, everyone interacts with the same gamification elements). Users differ in both behavior and motivation; therefore, gamified experiences should be tailored to their characteristics and contexts to maximize gamification effects [68-71]. For example, 2 training tasks may be gamified through the incorporation of a theme; however, the theme should be adapted to suit the target audience. In the study of Boendermaker et al [55], it is clear that the lighthearted “boy-meets-girl” love story theme is suitable for adolescents, but it may not necessarily be appropriate for children and adults. To date, the study of *personalized* gamification, while emerging, is still largely underexplored, and more research is needed to determine how best to satisfy individual and group differences in an effective way [72-75].

#### Cognitive Domains Targeted

In the systematic review, all training tasks were designed to improve various aspects of cognition. About two-thirds (n=31/49, 63%) of the studies conducted single-domain cognitive training, with the most targeted domain being attention (n=11/49, 22%), closely followed by working memory (n=9/49, 18%). This may be due to the fact that attentional processes and working memory are critical aspects of our cognitive capacities [76] that can be improved and modified with training [77,78]. The remaining assessed studies trained multiple cognitive domains simultaneously, making it difficult to isolate the impact of gamification on a specific domain. Overall, these findings are largely consistent with the results of Lumsden et al [10] who found gamified cognitive training interventions focused on one or two domains exclusively, mostly working memory.

#### Intervention Characteristics

In the systematic review, the type of technology used for delivering training tasks varied across studies, with computers (ie, desktop, laptop, and notebook) being the most widely used device (n=26/49, 53%), followed by tablets (n=13/49, 27%). Remarkably, only 2 studies employed smartphones [45,79], showing that the application of this technology to gamified cognitive training is still in its infancy. Unsurprisingly, therefore, training was mostly (n=19/49, 39%) delivered in a more controlled environment like the laboratory, hospital, or school. Of note, there is a lack of studies directly comparing the effects of the same intervention across different settings or delivery methods. To better understand the specific conditions for effective dissemination of gamified cognitive training interventions, future studies should investigate whether delivering training remotely and via mobile platforms (eg, smartphones, tablets) is acceptable and feasible. For example, it would be interesting to compare training outcomes (ie, cognitive and clinical) and adherence rates of a remote smartphone-based gamified intervention with an in-person desktop-based intervention in a randomized trial.

Overall, the number of training sessions ranged from only one session to a maximum of 59 sessions, for periods ranging from 5 min ([47], study 1) [54] up to 80 min [35] per training session. Previous research on cognitive training has shown a dose-response relationship between the number of training sessions and cognitive gains [80]. However, in the current meta-analysis, the number of sessions did not predict cognitive effects. It is possible that, in the context of gamification, more training sessions are needed, as there might be an *adaptation* time required to become used to the gamified environment. Meta-regression analyses also revealed no significant effect of the number of training sessions on effect sizes for motivation/engagement outcomes. The nonsignificant result of this moderator for cognitive process and motivation/engagement outcomes could be explained by the limited variability in the number of training sessions (Multimedia Appendix 3), making it difficult to detect a dose-response relationship.

#### Implementation of Gamification

In the systematic review, a broad range of game features have been integrated into training tasks, with the most frequent provision of elements involving achievement and progression-oriented features (see Table 1 for the full list). Specifically, a notable 82% (n=40/49) of all included studies incorporated rewards, such as points, badges, and stars, among others. This finding is consistent with previous systematic reviews [10,17], indicating that these features continue to dominate the landscape of gamification. Perhaps this is because rewards can easily be added as an additional layer to an existing paradigm without changing the original structure [81]. However, their use is not without critique, with researchers arguing that reward-based systems are motivating extrinsically, yet not intrinsically [13,15] with associated concerns regarding the longevity of any effects. Feedback loops were also frequently (n=39/49, 80%) employed as a means to amplify or reduce an action, and were delivered through various modalities, such as auditory (eg, high-pitched “Huh?” [82]), visual (eg, growing neuron animation [18]), and tactile (eg, vibration on the smartphone [79]). This observation is unsurprising because feedback is a key technique for behavior change [83] and can facilitate intrinsic motivation [84].

The second most frequently implemented category of game elements involves immersion-oriented features, such as story/theme, sound effects, and avatars (Table 1). In particular, the use of a story/theme was found to be high, with more than three-fourth of studies (n=38/49) using this technique to enhance the appeal of cognitive tasks. In some studies, it was evident that the theme was carefully selected to fit the target audience. For example, Dassen et al [85] presented their working memory tasks in a restaurant setting to help overweight adults improve self-regulation and increase weight loss. The high frequency of immersion-related features is encouraging because, from an SDT perspective [12,13], such elements can potentially satisfy the need for autonomy and drive intrinsic motivation [86]. On the other hand, social-oriented features were underutilized, with only a few studies favoring competition (n=3/49, 6%) and social interaction (n=2/49, 4%). Similar findings have been reported in a recent review investigating game-based interventions for neuropsychological assessment, training, and rehabilitation [87]. This may be due to implementation complexity or the relative recency of the field.

It is clear from our review that studies are driven by the presumption that gamification in cognitive training consists of embedding a combination of game elements within the training tasks. Indeed, no study has reported on the effect of a single element alone and the mode count of game elements was 5 (range 2-9). This figure is much higher than from a previous review of web-based mental health interventions (mode 1; range 1-3) [88], but is in line with a recent review of mental health and well-being apps (mode 5; range 1-11) [89], highlighting that the nature of gamification in the context of health is changing, becoming increasingly complex and diverse.

In the meta-analysis, we attempted to include the type of game elements used as a moderator; however, there were not enough studies in the subgroups for a conclusive evaluation (Tables 2 and 3). Meta-regression analyses revealed no significant effect of the number of game elements on effect sizes for motivation/engagement and cognitive process outcomes. This observation raises the question of whether there is a minimum number of gamification features that need to be implemented to achieve the threshold for motivation/engagement (without significantly impacting cognitive processes). A related question is when a gamified task should be considered to have become a game—the boundaries between gamification and serious games remain blurry and highly subjective [10,17,89].

With regard to theories of motivation, only around one-third of studies (n=17/49, 35%) in our systematic review mentioned using theoretical foundations to guide the development of their interventions. SDT [12,13], flow theory [50], and the framework by Kiili and Perttula [51] were among the most prominent choices of frameworks (n=7/49, 14%) used in gamified cognitive training. Of note, several studies (n=8/49, 16%) drew upon multiple theories and principles to design their training tasks. This lack of theoretical underpinning has been reported previously [14,17], calling for more theory-driven research on gamification in the field of health.

### Limitations

There are limitations to this study. First, the results of the systematic review and meta-analysis are limited by the relatively low methodological quality of retrieved studies, mostly due to inadequate or unclear blinding of participants and personnel. Previous reviews [17] have provided similar findings, reflecting the relative infancy of the gamification field in the health context. There was also a large degree of heterogeneity between studies including study design, target population, and outcome measures, which created challenges in synthesizing the literature and may have contributed to the heterogeneity detected in the meta-analyses. Of particular concern was the widespread use of self-developed, unvalidated tools to assess motivation and engagement outcomes. Thus, to facilitate interpretation of results and to advance gamification research, there is a need for experts and researchers to develop valid, reliable, and sensitive measures of motivation and engagement that are grounded in motivational theory and are applicable to the particular research and clinical context. Another limitation of the review was the modest number of studies (n=9/49, 18%) that we could include in the meta-analyses. In addition, sample sizes were rather small, especially for several process-related outcomes and subgroup analyses, limiting the generalizability of our results. Owing to the limited number of RCTs, we were also unable to test for the cognitive domain and type of game elements as potential moderators of gamification effects. The size and direction of the effects of gamification on cognitive outcomes may differ by domain; the examination of which requires more RCTs to be conducted in the domains of attention and inhibition. It is possible that the absence of evidence of any effect of gamification on cognitive outcomes overall might reflect the relatively small number of heterogeneous studies and not that gamification cannot be applied in a manner that will improve cognitive outcomes in particular domains. With the addition of further studies and associated variability, future meta-analyses may want to assess the influence of such moderators and potential sources of bias (eg, funding source) on review results and conclusions. Available study findings also did not address the long-term or sustained effects of gamified interventions due to limited follow-up assessments of outcomes (n=1/9, 11%) [54]. Finally, as mentioned previously, game elements were not always well-described in studies and investigated only in combination, making it impossible to establish whether individual elements had measurable effects. 

### Recommendations for Future Research

#### Exploration of Game Elements

Our review revealed that a wide array of game elements is being used; however, certain features (eg, rewards) continue to receive more attention than others (eg, social interactions). The further development of gamified tasks may benefit from drawing from the entire repertoire of game elements and tailoring gamification according to the users’ individual characteristics and contexts. Furthermore, consistent with previous reviews [17], there is a lack of research isolating the impact of single game elements. However, given that in real-world settings, gamification elements are not typically deployed in isolation, it may be most beneficial for future research to explore both the impact of individual elements as well as groups of elements.

#### Reporting of Gamification

Much progress can be made in improving the reporting of gamification elements, with only a few studies in our review explicitly describing how each element is operationalized in the interventions. As such, we propose the creation of reporting guidelines that outline a full description of the game elements used (via text and picture) in research, how they are being implemented, and what they aim to target. To facilitate understanding of which game elements are used and how they are operationalized, researchers should enable the scientific community to consult a (sample) version of the gamified task. This will improve the quality of gamified health-related publications and facilitate more informative systematic reviews.

#### Reviewing the Quality of Gamification

Relatedly, not only the reporting but also the evaluation of the quality of the operationalization of gamification is important. Therefore, we call for gamification developers to create guidelines for evaluating the quality of the intervention. Given that it is unlikely that such an evaluation can be made based on the information provided in the paper, gamified tasks should be more readily accessible to other researchers. Future research could serve the field well by *archiving* gamified interventions and by implementing effective data management strategies that use the Findable, Accessible, Interoperable, and Reusable principles [90].

#### Perceptions Toward Gamification

Surprisingly, almost no studies have investigated perceptions of different populations regarding the use of gamification. Indeed, while several studies have indicated that participants experience traditional cognitive training tasks as monotonous and boring [4,5], more research is needed to evaluate participants’ perceptions and readiness for gamification before determining whether it should be implemented. One way of achieving this is by using a combination of quantitative and qualitative methods to determine the target population’s perceptions and experiences of gamification as well as its receptiveness to using gamification in such tasks. This approach could also improve tailoring gamification to specific target groups. For example, adolescents (generation Z) and young adults (millennials) may be more responsive to gamification experience given their familiarity with a broad range of digital technologies.

#### Theory-Driven Research

Most reviewed studies lack a theoretical underpinning for the choice and design of their interventions. This issue is not new and has been reported previously [14,17], calling for more theory-driven research on gamification in the field of cognition and health more generally. Future research should develop and establish more formal working models of gamification that help understand *why* and *how* particular game elements work (or not work), when they work best, and the kind of effects they expect to have on psychological or behavioral outcomes.

#### Study Design and Reporting

Despite growing interest in applying gamification to cognitive training, there is still a lack of long-term follow-up and well-controlled studies in this field. More rigorously designed RCTs comparing gamified and nongamified versions of the same intervention, with adequately powered sample sizes and longer-term follow-up are needed. We also urge researchers to preregister research protocols and conform to the recommendations of the Consolidated Standards of Reporting Trials guidelines [91].

## Appendix

Multimedia Appendix 1 Literature search strategies.

Multimedia Appendix 2 Systematic review tables.

Multimedia Appendix 3 Meta-analysis tables and figures.

## Abbreviations

ADHD: attention-deficit/hyperactivity disorder
CMA: Comprehensive Meta-Analysis
RCT: randomized controlled trial
SDT: self-determination theory

## References

[1] Harvey PD, McGurk SR, Mahncke H, Wykes T (2018). Controversies in computerized cognitive training. Biol Psychiatry Cogn Neurosci Neuroimaging.

[2] Lampit A, Hallock H, Valenzuela M (2014). Computerized cognitive training in cognitively healthy older adults: a systematic review and meta-analysis of effect modifiers. PLoS Med.

[3] Kueider AM, Parisi JM, Gross AL, Rebok GW (2012). Computerized cognitive training with older adults: a systematic review. PLoS One.

[4] Beard C, Weisberg RB, Primack J (2012). Socially anxious primary care patients' attitudes toward cognitive bias modification (CBM): a qualitative study. Behav Cogn Psychother.

[5] Kuckertz JM, Schofield CA, Clerkin EM, Primack J, Boettcher H, Weisberg RB, Amir N, Beard C (2019). Attentional bias modification for social anxiety disorder: what do patients think and why does it matter?. Behav Cogn Psychother.

[6] Jaeggi SM, Buschkuehl M, Shah P, Jonides J (2014). The role of individual differences in cognitive training and transfer. Mem Cognit.

[7] Redick TS, Shipstead Z, Harrison TL, Hicks KL, Fried DE, Hambrick DZ, Kane MJ, Engle RW (2013). No evidence of intelligence improvement after working memory training: a randomized, placebo-controlled study. J Exp Psychol Gen.

[8] Katz B, Jaeggi S, Buschkuehl M, Stegman A, Shah P (2014). Differential effect of motivational features on training improvements in school-based cognitive training. Front Hum Neurosci.

[9] Fleming T, Dixon R, Frampton C, Merry S (2012). A pragmatic randomized controlled trial of computerized CBT (SPARX) for symptoms of depression among adolescents excluded from mainstream education. Behav Cogn Psychother.

[10] Lumsden J, Edwards EA, Lawrence NS, Coyle D, Munafò MR (2016). Gamification of cognitive assessment and cognitive training: a systematic review of applications and efficacy. JMIR Serious Games.

[11] Deterding S, Dixon D, Khaled R, Nacke L (2011). From Game Design Elements to Gamefulness: Defining 'Gamification'. Proceedings of the 15th International Academic MindTrek Conference: Envisioning Future Media Environments.

[12] Deci EL, Ryan RM (1985). Intrinsic Motivation and Self-Determination in Human Behavior.

[13] Ryan RM, Deci E (2000). Intrinsic and extrinsic motivations: classic definitions and new directions. Contemp Educ Psychol.

[14] Seaborn K, Fels DI (2015). Gamification in theory and action: a survey. Inte JHum-Comput Stud.

[15] Nicholson S, Reiners T, Wood LC (2015). A RECIPE for meaningful gamification. Gamification in Education and Business.

[16] Muntean CI (2011). Raising Engagement in E-Learning Through Gamification. Proceedings of the 6th International Conference on Virtual Learning.

[17] Johnson D, Deterding S, Kuhn K, Staneva A, Stoyanov S, Hides L (2016). Gamification for health and wellbeing: a systematic review of the literature. Internet Interv.

[18] Ninaus M, Pereira G, Stefitz R, Prada R, Paiva A, Neuper C, Wood G (2015). Game elements improve performance in a working memory training task. Int J Serious Games.

[19] Lee T, Goh SJ, Quek SY, Phillips R, Guan C, Cheung YB, Feng L, Teng SS, Wang CC, Chin ZY, Zhang H, Ng TP, Lee J, Keefe R, Krishnan KR (2013). A brain-computer interface based cognitive training system for healthy elderly: a randomized control pilot study for usability and preliminary efficacy. PLoS One.

[20] Moher D, Liberati A, Tetzlaff J, Altman DG, PRISMA Group (2009). Preferred reporting items for systematic reviews and meta-analyses: the PRISMA statement. PLoS Med.

[21] Johnson D, Horton E, Mulcahy R, Foth M (2017). Gamification and serious games within the domain of domestic energy consumption: a systematic review. Renew Sust Energ Rev.

[22] Sardi L, Idri A, Fernández-Alemán JL (2017). A systematic review of gamification in e-health. J Biomed Inform.

[23] Higgins J, Li T, Deeks J, Higgins J, Thomas J, Chandler J, Cumpston M, Page MJ, Welch VA (2019). Choosing effect measures and computing estimates of effect. Cochrane Handbook for Systematic Reviews of Interventions. Second Edition.

[24] van Ryckeghem DM, van Damme S, Eccleston C, Crombez G (2018). The efficacy of attentional distraction and sensory monitoring in chronic pain patients: a meta-analysis. Clin Psychol Rev.

[25] Prins PJ, Dovis S, Ponsioen A, ten Brink E, van der Oord S (2011). Does computerized working memory training with game elements enhance motivation and training efficacy in children with ADHD?. Cyberpsychol Behav Soc Netw.

[26] Higgins J, Altman D, Sterne J, Higgins JP, Green S (2011). Assessing risk of bias in included studies. Cochrane Handbook for Systematic Reviews of Interventions.

[27] Koivisto J, Hamari J (2014). Demographic differences in perceived benefits from gamification. Comput Hum Behav.

[28] Koivisto J, Hamari J (2019). The rise of motivational information systems: a review of gamification research. Int J Inf Manag.

[29] Hedges L, Olkin I (1985). Statistical Methods for Meta-Analysis.

[30] Borenstein M, Hedges L, Higgins J, Rothstein H (2009). Introduction to Meta-Analysis.

[31] Thompson SG, Higgins JP (2002). How should meta-regression analyses be undertaken and interpreted?. Stat Med.

[32] Egger M, Smith GD, Schneider M, Minder C (1997). Bias in meta-analysis detected by a simple, graphical test. Br Med J.

[33] Duval S, Tweedie R (2000). Trim and fill: a simple funnel-plot-based method of testing and adjusting for publication bias in meta-analysis. Biometrics.

[34] Cohen J (1988). Statistical Power Analysis for the Behavioral Sciences. Second Edition.

[35] Brezovszky B, Lehtinen E, McMullen J, Rodriguez G, Veermans K (2013). Training Flexible and Adaptive Arithmetic Problem Solving Skills Through Exploration With Numbers: the Development of Numbernavigation Game. Proceedings of the 7th European Conference on Games Based Learning.

[36] Connor B, Shaw C (2016). Case study series using brain-training games to treat attention and memory following brain injury. J Pain Manag.

[37] Double KS, Birney DP (2016). The effects of personality and metacognitive beliefs on cognitive training adherence and performance. Pers Individ Differ.

[38] Jaeggi SM, Buschkuehl M, Jonides J, Shah P (2011). Short- and long-term benefits of cognitive training. Proc Natl Acad Sci U S A.

[39] Nagle A, Novak D, Wolf P, Riener R (2015). Increased enjoyment using a tablet-based serious game with regularly changing visual elements: a pilot study. Gerontechnology.

[40] De Vries M, Prins PJ, Schmand BA, Geurts HM (2015). Working memory and cognitive flexibility-training for children with an autism spectrum disorder: a randomized controlled trial. J Child Psychol Psychiatry.

[41] Hiraoka T, Wang T, Kawakami H (2016). Cognitive function training system using game-based design for elderly drivers. IFAC-PapersOnLine.

[42] Dennis-Tiwary TA, Denefrio S, Gelber S (2017). Salutary effects of an attention bias modification mobile application on biobehavioral measures of stress and anxiety during pregnancy. Biol Psychol.

[43] Boletsis C, McCallum S (2016). Augmented reality cubes for cognitive gaming: preliminary usability and game experience testing. Int J Serious Games.

[44] Enock P (2015). Making an IMPACT: Designing and Testing a Novel Attentional Training Game to Reduce Social Anxiety. Harvard University.

[45] Garolera M, Berga N, Quintana M, Chico G, Cerulla N, López M, Donaire Y, Rimbau J (2015). Active-U: Playing to Stimulate your Brain. Proceedings of the 2nd International Workshop on Gamification in Health.

[46] Abellanoza C (2017). Rewind-Remind: Investigating How Gamification of Memory Tasks Can Evaluate Associative Memory Performance in Healthy, Older Adults. The University of Texas at Arlington.

[47] Boendermaker WJ, Boffo M, Wiers RW (2015). Exploring elements of fun to motivate youth to do cognitive bias modification. Games Health J.

[48] Trapp W, Landgrebe M, Hoesl K, Lautenbacher S, Gallhofer B, Günther W, Hajak G (2013). Cognitive remediation improves cognition and good cognitive performance increases time to relapse--results of a 5 year catamnestic study in schizophrenia patients. BMC Psychiatry.

[49] Birk MV, Mandryk RL, Atkins C (2016). The Motivational Push of Games: The Interplay of Intrinsic Motivation and External Rewards in Games for Training. Proceedings of the 2016 Annual Symposium on Computer-Human Interaction in Play.

[50] Csikszentmihalyi M (1990). Flow: The Psychology of Optimal Experience.

[51] Kiili K, Perttula A, Freitas S, Ott M, Popescu MM, Stanescu I (2013). A design framework for educational exergames. New Pedagogical Approaches in Game Enhanced Learning: Curriculum Integration.

[52] Gee JP (2016). Learning by design: good video games as learning machines. E-Learn Digit Media.

[53] Mohammed S, Flores L, Deveau J, Hoffing RC, Phung C, Parlett CM, Sheehan E, Lee D, Au J, Buschkuehl M, Zordan V, Jaeggi SM, Seitz AR (2017). The benefits and challenges of implementing motivational features to boost cognitive training outcome. J Cogn Enhanc.

[54] Boendermaker WJ, Maceiras SS, Boffo M, Wiers RW (2016). Attentional bias modification with serious game elements: evaluating the shots game. JMIR Serious Games.

[55] Boendermaker WJ, Veltkamp RC, Peeters M (2017). Training behavioral control in adolescents using a serious game. Games Health J.

[56] Choi J, Medalia A (2010). Intrinsic motivation and learning in a schizophrenia spectrum sample. Schizophr Res.

[57] Dörrenbächer S, Müller PM, Tröger J, Kray J (2014). Dissociable effects of game elements on motivation and cognition in a task-switching training in middle childhood. Front Psychol.

[58] Lopez CE, Tucker CS (2017). A quantitative method for evaluating the complexity of implementing and performing game features in physically-interactive gamified applications. Comput Hum Behav.

[59] Bogost I, Walz SP, Deterding S (2015). Why gamification is bullshit. The Gameful World: Approaches, Issues, Applications.

[60] Rojas D, Kapralos B, Dubrowski A (2013). The Missing Piece in the Gamification Puzzle. Proceedings of the First International Conference on Gameful Design, Research, and Applications.

[61] Wen C, Hsien H, Huang H (2015). A Survey of Gamification for Healthcare System. Proceedings of the International Conference on Orange Technologies.

[62] Craig P, Dieppe P, Macintyre S, Michie S, Nazareth I, Petticrew M, Medical Research Council Guidance (2008). Developing and evaluating complex interventions: the new medical research council guidance. Br Med J.

[63] Campbell M, Fitzpatrick R, Haines A, Kinmonth A, Sandercock P, Spiegelhalter D, Tyrer P (2000). Framework for design and evaluation of complex interventions to improve health. Br Med J.

[64] Kiili K, Ninaus M, Koskela M, Tuomi M, Lindstedt A (2013). Developing Games for Health Impact: Case Brains vs Zombies. Proceedings of the 7th European Conference on Games Based Learning.

[65] Dennis-Tiwary TA, Egan LJ, Babkirk S, Denefrio S (2016). For whom the bell tolls: neurocognitive individual differences in the acute stress-reduction effects of an attention bias modification game for anxiety. Behav Res Ther.

[66] van der Oord S, Ponsioen AJ, Geurts HM, ten Brink EL, Prins PJ (2014). A pilot study of the efficacy of a computerized executive functioning remediation training with game elements for children with ADHD in an outpatient setting: outcome on parent- and teacher-rated executive functioning and ADHD behavior. J Atten Disord.

[67] Notebaert L, Clarke PJ, Grafton B, MacLeod C (2015). Validation of a novel attentional bias modification task: the future may be in the cards. Behav Res Ther.

[68] Kappen D, Mirza-Babaei P, Nacke L (2017). Gamification through the Application of Motivational Affordances for Physical Activity Technology. Proceedings of the Annual Symposium on Computer-Human Interaction in Play.

[69] Codish D, Ravid G (2014). Personality Based Gamification- Educational Gamification for Extroverts and Introverts. Proceedings of the 9th CHAIS Conference for the Study of Innovation and Learning Technologies: Learning in the Technological Era.

[70] Orji R, Mandryk R, Vassileva J, Gerling K (2013). Tailoring Persuasive Health Games to Gamer Type. Proceedings of the SIGCHI Conference on Human Factors in Computing Systems.

[71] Böckle M, Novak J, Bick M (2017). Towards Adaptive Gamification: A Synthesis of Current Developments. Proceedings of the 25th European Conference on Information Systems.

[72] Knutas A, van Roy R, Hynninen T, Granato M, Kasurinen J, Ikonen J (2018). A process for designing algorithm-based personalized gamification. Multimed Tools Appl.

[73] Oliveira W, Bittencourt I (2019). Research advances on tailored gamification. Tailored Gamification to Educational Technologies.

[74] Tondello G, Wehbe R, Diamond L, Busch M, Marczewski A, Nacke L (2016). The Gamification User Types Hexad Scale. Proceedings of the 2016 Annual Symposium on Computer-Human Interaction in Play.

[75] Hallifax S, Serna A, Marty J, Lavoué G, Lavoué E (2019). Factors to Consider for Tailored Gamification. Proceedings of the Annual Symposium on Computer-Human Interaction in Play.

[76] Fougnie D, Johansen NB (2008). The relationship between attention and working memory. New Research on Short-Term Memory.

[77] Zhang H, Huntley J, Bhome R, Holmes B, Cahill J, Gould RL, Wang H, Yu X, Howard R (2019). Effect of computerised cognitive training on cognitive outcomes in mild cognitive impairment: a systematic review and meta-analysis. BMJ Open.

[78] Hill NT, Mowszowski L, Naismith SL, Chadwick VL, Valenzuela M, Lampit A (2017). Computerized cognitive training in older adults with mild cognitive impairment or dementia: a systematic review and meta-analysis. Am J Psychiatry.

[79] Lukas CA, Berking M (2018). Reducing procrastination using a smartphone-based treatment program: a randomized controlled pilot study. Internet Interv.

[80] Jaeggi SM, Buschkuehl M, Jonides J, Perrig WJ (2008). Improving fluid intelligence with training on working memory. Proc Natl Acad Sci U S A.

[81] Boendermaker WJ, Prins PJ, Wiers RW (2015). Cognitive bias modification for adolescents with substance use problems--can serious games help?. J Behav Ther Exp Psychiatry.

[82] Dennis TA, O'Toole L (2014). Mental health on the go: effects of a gamified attention bias modification mobile application in trait anxious adults. Clin Psychol Sci.

[83] Cugelman B (2013). Gamification: what it is and why it matters to digital health behavior change developers. JMIR Serious Games.

[84] Hicks K, Gerling K, Richardson G, Pike T, Burman O, Dickinson P (2019). Understanding the Effects of Gamification and Juiciness on Players. Proceedings of the IEEE Conference on Games.

[85] Dassen FC, Houben K, van Breukelen GJ, Jansen A (2018). Gamified working memory training in overweight individuals reduces food intake but not body weight. Appetite.

[86] Xi N, Hamari J (2019). Does gamification satisfy needs? A study on the relationship between gamification features and intrinsic need satisfaction. Int J Inf Manag.

[87] Ferreira-Brito F, Fialho M, Virgolino A, Neves I, Miranda AC, Sousa-Santos N, Caneiras C, Carriço L, Verdelho A, Santos O (2019). Game-based interventions for neuropsychological assessment, training and rehabilitation: which game-elements to use? A systematic review. J Biomed Inform.

[88] Brown M, O'Neill N, van Woerden H, Eslambolchilar P, Jones M, John A (2016). Gamification and adherence to web-based mental health interventions: a systematic review. JMIR Ment Health.

[89] Cheng VW, Davenport T, Johnson D, Vella K, Hickie IB (2019). Gamification in apps and technologies for improving mental health and well-being: systematic review. JMIR Ment Health.

[90] Wilkinson MD, Dumontier M, Aalbersberg IJ, Appleton G, Axton M, Baak A, Blomberg N, Boiten J, da Silva LB, Bourne PE, Bouwman J, Brookes AJ, Clark T, Crosas M, Dillo I, Dumon O, Edmunds S, Evelo CT, Finkers R, Gonzalez-Beltran A, Gray AJ, Groth P, Goble C, Grethe JS, Heringa J, 't Hoen PA, Hooft R, Kuhn T, Kok R, Kok J, Lusher SJ, Martone ME, Mons A, Packer AL, Persson B, Rocca-Serra P, Roos M, van Schaik R, Sansone S, Schultes E, Sengstag T, Slater T, Strawn G, Swertz MA, Thompson M, van der Lei J, van Mulligen E, Velterop J, Waagmeester A, Wittenburg P, Wolstencroft K, Zhao J, Mons B (2016). The FAIR guiding principles for scientific data management and stewardship. Sci Data.

[91] Schulz KF, Altman DG, Moher D, CONSORT Group (2010). CONSORT 2010 statement: updated guidelines for reporting parallel group randomised trials. BMC Med.

