# Supplementary material for: The Effects of Gamification on Computerized Cognitive Training: Systematic Review and Meta-Analysis
Source: JMIR Serious Games. 2020 Aug 10;8(3):e18644. doi: 10.2196/18644 (PMC7445616; doi:10.2196/18644)
Supplement: Multimedia Appendix 1 [file games_v8i3e18644_app1.pdf]

Multimedia Appendix 1: Literature Search Strategies

We employed for each database full and truncated search terms in the following search string:

Gamification: (gamif* OR "game design element*" OR "game-design element*" OR "game element*" OR "game design interface pattern*" OR "game-design interface pattern*" OR "game interface element*" OR "game mechanic*" OR "game feature*" OR "game-like element*" OR "game-like feature*" OR "videogame element*") AND

Cognition: (cognit* OR "mental process*" OR attention* OR memory OR inhibition OR "inhibitory control" OR "impulse control" OR "executive function*" OR "mental planning" OR "cognitive planning" OR "planning ability" OR "decision making" OR "problem solving" OR "visual perception" OR "auditory perception" OR "tactile perception" OR "processing speed" OR "psychomotor speed" OR "psycho-motor speed" OR set-shifting OR "mental flexibility" OR "cognitive flexibility") AND

Training: (train* OR modif* OR chang* OR intervention* OR treat* OR therap*).

Although the search strategy was the same for each database, suitable changes were made to accommodate for the different interfaces.

<u>Example of a search strategy:</u>

Database: PscycINFO

Host: EBSCOhost

Date searched:14/2/2018

Hits: 82

Strategy: (gamif* OR "game design element*" OR "game-design element*" OR "game element*" OR "game design interface pattern*" OR "game-design interface pattern*" OR "game interface element*" OR "game mechanic*" OR "game feature*" OR "game-like element*" OR "game-like feature*" OR "videogame element*") AND (cognit* OR "mental

process*” OR attention* OR memory OR inhibition OR “inhibitory control” OR “impulse control” OR “executive function*” OR “mental planning” OR “cognitive planning” OR “planning ability” OR “decision making” OR “problem solving” OR “visual perception” OR “auditory perception” OR “tactile perception” OR “processing speed” OR “psychomotor speed” OR “psycho-motor speed” OR set-shifting OR “mental flexibility” OR “cognitive flexibility”) AND (train* OR modif* OR chang* OR intervention* OR treat* OR therap*)
