# Supplementary material for: The Effects of Gamification on Computerized Cognitive Training: Systematic Review and Meta-Analysis
Source: JMIR Serious Games. 2020 Aug 10;8(3):e18644. doi: 10.2196/18644 (PMC7445616; doi:10.2196/18644)
Supplement: Multimedia Appendix 2 [file games_v8i3e18644_app2.pdf]

Multimedia Appendix 2: Systematic Review Tables

Table 2.1. Summary descriptions of studies included in the systematic review in alphabetical order.

Author (year)	Publication type; country	Cognitive domain	Study design	Participant characteristics				Intervention characteristics				Gamification characteristics			Outcomes
				Type	*N*	M_{age}	% female	Study setting	Study device	Study duration; follow-up	*N* session; session length	Game name	Game element	Theory used	
Abellanoza (2017)	PhD thesis; United States	Associative memory	Between-subject (pre-post) design; 6 groups (younger adults [control, directed encoding, self-generated encoding], older adults [control, directed encoding, self-generated encoding])	Younger and older adults	80	40.43	58	Home	Computer or laptop	3 weeks; none	9; minimum 10-15 min	Rewind-Remind	Feedback loops, progress, story/theme	NS	Associative memory, noun-pair lookup table usage, general cognitive ability, everyday cognitive ability, user attitudes, opinions and playability
Baniqued et al. (2015)	Published article; United States	Inductive reasoning, reasoning/Gf, task-switching, visuospatial reasoning, WM	Between-subject (pre-post) design; 2 groups (Mind Frontiers, active control)	Adults between 18 and 30 years old	90	21.00	57	Laboratory	Portable handheld devices	4-5 weeks; none	20; 72 min	Mind Frontiers	Challenge, DDA, feedback loops, game level, progress, rewards, story/theme	NS	Reasoning/fluid intelligence, WM, perceptual speed, episodic memory, selective attention, divided attention, multi-tasking, training feedback (enjoyment, engaging, demanding/effortful, motivation, frustration)
Birk et al. (2016)	Conference paper; Canada	EFs	Between-subject (pre-post) design; 2 groups (less-identified, more-identified)	MTurk adults	170	31.70 [a]	44 [a]	Online	NS	11 days; none	11; 10 min	Zombie Apocalypse	Avatar (assigned or created), feedback loops, rewards, story/theme	SDT	Task performance, motivation (participation rates, enjoyment, effort-importance, perceived competence, tension-pressure)
Boendermaker et al. (2015)- Study 1*	Published article; the Netherlands	Inhibition (alcohol)	Between-subject (pre-post) design; 4 groups (GNG original training, GNG placebo, GNG game, GNG social game)	Regular drinking undergraduate students	70	22.70 [a]	51 [a]	Laboratory or home	Computer	5 days-2 weeks; 1 week	3; 5-10 min	Cheese Ninja Game	Avatar, challenge, competition, game level, progress, rewards, social interaction, sound effects, story/theme	NS	Alcohol-related memory bias, alcohol use, motivation to train, user experience (ease of use, task immersion, task demand, task clarity)
Boendermaker et al. (2016)*	Published article; the Netherlands	Attention (alcohol)	Between-subject (pre-post) design; 3 groups (VPT regular, VPT placebo, VPT gamified)	Heavy drinking undergraduate students	94	21.20 [a]	71 [a]	Laboratory and home	Computer	2 weeks; 2 weeks	4; 5-10 min	Shots Game	Feedback loops, game level, progress, rewards, sound effects, story/theme	NS	Alcohol attentional bias, alcohol consumption, binge drinking, motivation to change, motivation to train
Boendermaker et al. (2017)*	Published article; the Netherlands	Response inhibition	Between-subject (pre-post) design; 3 groups (non-game training, game placebo, game training)	Adolescents in secondary education vocational tracks	173	14.90[a]	57 [a]	School	Computer or laptop	4 weeks; 4-6 weeks	4; 10-15 min	The Fling	Feedback loops, game level, rewards, sound effects, story/theme	NS	Behavioural control, WM capacity, drinking behaviour, motivation/appreciation
Boletsis and McCallum (2016)	Published article; Norway	Attention, EFs (flexibility, response inhibition, problem solving, decision making, WM), language processing, perception, spatial processing, visual processing	Single group design	Healthy older adults	5	67.60	NS	Laboratory	Tablet	45-55 min; none	1; 45-55 min	CogARC	Challenge, competition, feedback loops, game level, rewards	NS	In-game experience, user experience
Boot et al. (2016)	Conference paper; United States	Inductive reasoning, planning, spatial reasoning, speed processing, task-switching, WM	Between-subject (post) design; 2 groups (brain training games, control games)	Older USA adults	60	72.35	57	Home	Tablet	1 month; none	30; 45 min	Mind Frontiers	Challenge, DDA, feedback loops, game level, progress, rewards, story/theme	Unified theory of acceptance and use of technology, technology acceptance model	Intervention expectations (vision, reaction time, memory, hand-eye coordination, reasoning, multi-tasking ability, ability to perform everyday tasks), intervention motivation, game perception (enjoyable, challenging, frustrating), adherence
Brezovszky et al. (2013)	Conference paper; Finland	Arithmetic problem solving flexibility	Case study	Elementary school children	3	10.33	0	NS	Computer	60-80 min; none	1; 60-80 min	NumberNavigation Game	Feedback loops, social interaction, story/theme	NS	Game patterns, in-game arithmetic flexibility, game experience, engagement

Author (year)	Publication type; country	Cognitive domain	Study design	Participant characteristics				Intervention characteristics				Gamification characteristics			Outcomes
				Type	*N*	M_{age}	% female	Study setting	Study device	Study duration; follow-up	*N* session; session length	Game name	Game element	Theory used	
Choi and Medalia (2010)*	Published article; United States	Arithmetic ability	Between-subject (pre-post) design; 3 groups (Motivational Math Game, math game, non-trained)	Outpatients with schizophrenia or schizoaffective disorder	72	39.05	33	Hospital	Computer	4 weeks; none	10; 30 min	Motivational Math Game	Avatar, competition, manual difficulty adjustment, story/theme	SDT, three-pronged motivational approach by Cordova and Lepper (1996)	Arithmetic skill, attention, perceived self-competency, psychiatric symptoms, perception of treatment autonomy, treatment intensity, intrinsic motivation
Connor and Shaw (2016)	Published article; United States	Attention, flexibility, memory, problem solving, processing speed	Case study	Adults with acquired brain injury	3	53.30	33	Hospital and home (if they wanted)	Computer or tablet	12 weeks; none	Minimum 24; 30-40 min	Lumosity	Challenge, DDA, feedback loops, rewards, sound effects	NS	Psychosocial functioning, visual attention, visual memory, overall cognitive performance, overall impression of the game
Dassen et al. (2017)	Published article; the Netherlands	WM	Between-subject (post) design; 2 groups (gamified WM training, sham training)	Overweight adults with a desire to lose weight	67	47.97[a]	75 [a]	Home (online)	Computer or tablet	On average within 33.57 days (between 25 and 50 days); 1 and 6 months	20-25; 38.44 min	Gamified WM training	Challenge, DDA, rewards, story/theme	NS	Food intake, BMI, WM, executive functioning, self-control, eating psychopathology, eating style, healthy eating behaviour
De Vries et al. (2015)	Published article; the Netherlands	EFs (mental flexibility, WM)	Between-subject (pre-post) design; 3 groups (adaptive WM training, adaptive cognitive flexibility training, non-adaptive control training)	Children with autism spectrum disorder	90	10.56	9	Home	Laptop	6 weeks; 6 weeks	25; 45 min	Braingame Brian	Avatar, challenge, DDA, feedback loops, game level, progress, rewards, sound effects, story/theme	NS	WM, cognitive flexibility, inhibition, sustained attention, far-transfer to daily life (EFs), social behaviour, ADHD behaviour, quality of life
Dennis and O'Toole (2014)	Published article; United States	Attention (threat)	Between-subject (pre-post) design; 4 groups (short ABMT, short placebo training, long ABMT, long placebo training)	Highly trait anxious undergraduate students	76	21.26	71	Laboratory	iPod Touch	20 min (short training) or 45 min (long training); none	1; 20 min (short training) or 45 min (long training)	ABMTApp	Feedback loops, progress, rewards, sound effects, story/theme	NS	Threat attentional bias, threat bias (vigilance, disengagement), state anxiety, mood (stress reactivity), anxious behaviour (stress reactivity), nervous speech (stress reactivity)
Dennis-Tiwary et al. (2017)	Published article; United States	Attention (threat)	Between-subject (pre-post) design; 2 groups (ABMT, placebo training)	Women in their 19th-29th week of pregnancy	29	32.97	100	Home	iOS device or iPod Touch	Approximately 4 weeks; none	16; 10 min	Personal Zen App	Feedback loops, progress, rewards, sound effects, story/theme	NS	Threat attentional bias, threat bias (vigilance, disengagement), anxiety, stress, stress reactivity, anxious behaviour (stress reactivity), nervous speech (stress reactivity)
Dennis-Tiwary et al. (2016)	Published article; United States	Attention (threat)	Between-subject (pre-post) design; 2 groups (ABMT, placebo training)	Trait anxious adults	42	20.60	50	Laboratory	iPod Touch	45 min; none	1; 45 min	Personal Zen App	Feedback loops, progress, rewards, sound effects, story/theme	NS	Threat attentional bias, state anxiety, negative mood, observed stress-related behaviours
Dorrenbacher et al. (2014)*	Published article; Germany	Executive control (task-switching)	Between-subject (pre-post) design; 4 groups (single-task low-motivational, single-task high-motivational, task-switching low-motivational, task-switching high-motivational)	Middle-aged children	54	9.64	48	Laboratory	Notebook	2-4 weeks; none	4; 30-45 min	Watermons	Avatar, DDA, feedback loops, rewards, story/theme	SDT, self-determinative interpretation of the motivational incitement of video-games	Task performance, task-switching performance, near-transfer performance, far-transfer performance to inhibitory control, far-transfer performance on WM, motivation training willingness (intrinsic interest)
Double and Birney (2016)	Published article; Australia	Attention, general knowledge, memory, mental flexibility	Single group design	Older Australians	794	61.95	77	Online	Computer	18 months; none	Up to the participant; up to the participant	Active Memory	DDA, progress	NS	Training performance, training adherence
Dovis et al. (2015)	Published article; the Netherlands	EFs (inhibition, mental flexibility, WM)	Between-subject (pre-post) design; 3 groups (full-active, partially-active, full placebo)	Children with a clinical diagnosis of ADHD	81	10.50 [a]	20 [a]	Home	Computer	5 weeks; 3 months	25; 35-50 min	Braingame Brian	Avatar, challenge, DDA, feedback loops, game level, progress, rewards, sound effects, story/theme	NS	Visuospatial short-term memory, visuospatial WM, verbal short-term memory, WM, response inhibition, interference control, cognitive flexibility, complex reasoning, ADHD behaviour, EFs, motivational behaviour, general problem behaviour
Enock (2015)	PhD thesis; United Kingdom	Attention (social anxiety)	Between-subject (post) design; 3 groups (IMPACT attend positive, IMPACT attend negative, IMPACT undirected)	MTurk adults	569	NS	NS	Online	Smartphone, tablet, or computer	15 min; none	1; 15 min	IMPACT	DDA, feedback loops, rewards	Operant conditioning	General attentional control, emotional attentional control, attentional bias, anxiety reactivity, user experience

Author (year)	Publication type; country	Cognitive domain	Study design	Participant characteristics				Intervention characteristics				Gamification characteristics			Outcomes
				Type	*N*	M_{age}	% female	Study setting	Study device	Study duration; follow-up	*N* session; session length	Game name	Game element	Theory used	
Garolera et al. (2015)	Conference paper; Spain	Attention, speed of information processing, WM	Between-subject (post) design; 2 groups (Unlocked, Active-U)	Patients with mild cognitive impairments	17	NS	NS	NS	iPhone	NS; none	3; NS	Active-U	Avatar, challenge, game level, progress, rewards, story/theme	NS	Usability
Gehring et al. (2011)	Published article; the Netherlands	Attention	Single group design	Patients with gliomas	60	42.00 [a]	41 [a]	Home or hospital (offered the choice)	Notebook	6 weeks; 6 months	6; at least 45 min	Concentration Car	DDA, feedback loops, progress, rewards, story/theme	NS	Attrition, compliance, engagement, subjective evaluation of attention exercises
Gehring et al. (2009)	Published article; the Netherlands	Attention	Between-subject (pre-post) design; 2 groups (experimental, waiting-list control)	Patients with gliomas	127	42.90[a]	42[a]	Home or hospital (offered the choice)	Notebook	6 weeks; 6 months	6; at least 45 min	Concentration Car	DDA, feedback loops, progress, rewards, story/theme	NS	Attention, verbal memory, EFs, cognitive symptoms and functioning, mental health-related quality of life, mental aspects of fatigue, home integration, social integration, productivity, evaluation of the cognitive rehabilitation program
Hiraoka et al. (2016)	Conference paper; Japan	Attention	Single group design	Elderly drivers	11	69.82	0	Laboratory and home	Tablet	6 days spread over two to three weeks; none	10 in laboratory and freely at home without any constraints; NS	Trail Making Game	Challenge, DDA, game level, progress	SDT, cognitive evaluation theory	Useful field of view, hazard perception ability, training performance, user experience (fun, motivation, competence, understanding, appropriateness of difficulty, burden, comfort)
Jaeggi et al. (2011)	Published article; United States	WM	Between-subject (pre-post) design; 2 groups (experimental, active control)	Elementary and middle school children	62	8.98	47	NS	Computer	1 month; 3 months	20 (minimum 15); 15 min	NS	Challenge, DDA, feedback loops, game level, progress, rewards, story/theme	NS	Training performance, fluid intelligence, engagement/motivation (interest/enjoyment, difficult/effort, perceived competence)
Katz et al. (2014)*	Published article; United States	WM	Between-subject (pre-post) design; 7 groups (all optional features included, no theme change, no points shown, no prizes, no explanation of lives/levels, no explanation of lives/levels or certificates, no optional features included)	Students in second through eight grade	107	10.65	44	School-based summer camps	Laptop	3 days; none	3; 9-11 min	Cognitive training game	DDA, feedback loops, game level, rewards, story/theme	SDT	Training performance, near-transfer performance, task difficulty, task effort, task excitement, enjoyment, engagement
Kiili et al. (2013)-Study 1	Conference paper; Finland	WM	Single group design	University students	6	NS	83	NS	Tablet	10-20 min; none	1; 10-20 min	Brains vs. Zombies	Avatar, challenge, feedback loops, game level, manual difficulty adjustment, rewards, sound effects, story/theme	Kiili and Perttula (2013) framework	Player experience, playing behaviour
Kiili et al. (2013)-Study 2	Conference paper; Finland	WM	Single group design	Primary school children	14	NS	50	Primary school	Tablet	10-20 min; none	1; 10-20 min	Brains vs. Zombies	Avatar, challenge, feedback loops, game level, manual difficulty adjustment, rewards, sound effects, story/theme	Kiili and Perttula (2013) framework	Player experience, playing behaviour
Kühn et al. (2017)	Published article; Germany	Inhibition	Between-subject (pre-post) design; 3 groups (inhibition game, active control, passive control)	Healthy older adults	48	69.00 [a]	51 [a]	Home	Tablet	8 weeks; none	maximum 56; 15 min	Schiff Ahoi	Challenge, DDA, feedback loops, progress, rewards, sound effects, story/theme	NS	Structural plasticity, behavioural plasticity (response inhibition), functional neural plasticity
Lee et al. (2013)	Published article; Singapore	Attention, memory	Between-subject (pre-post) design; 2 groups (BCI intervention, wait-list control)	Healthy elderly	31	65.10	60	Laboratory	Computer	8 weeks; 2 months	24; 30 min	BCI Memory and Attention Training Game	Feedback loops, game level, rewards	NS	Immediate memory, delayed memory, language, visuospatial/constructional, attention, detecting and characterising dementia, usability and acceptability (satisfaction, comfort, enjoyment, usefulness, recommendation, satisfaction-interface, satisfaction-system), safety, adherence
Lukas and Berking (2017)	Published article; Germany	Approach bias (procrastination)	Between-subject (pre-post) design; 2 groups (MT-PRO training, waitlist)	Adults with heightened procrastination scores	31	22.03	84	Location of choice	Smartphone	14 days; 4 weeks	NS; NS	MT-PRO	Feedback loops, rewards	Operant conditioning, computer gaming principles	General procrastination symptoms, academic procrastination symptoms, motivation to change, emotion regulation skills, feedback regarding application

Author (year)	Publication type; country	Cognitive domain	Study design	Participant characteristics				Intervention characteristics				Gamification characteristics			Outcomes
				Type	*N*	M_{age}	% female	Study setting	Study device	Study duration; follow-up	*N* session; session length	Game name	Game element	Theory used	
Mohammed et al. (2017)*	Published article; United States	WM	Between-subject (pre-post) design; 2 groups (Tapback, Recall)	Undergraduate students	115	19.98	58	Laboratory	Tablet	4 weeks; none	20; 20 min	Recall the Game	Challenge, difficulty adjustment (dynamic and manual), feedback loops, game level, progress, rewards, sound effects, story/theme	Perceptual Learning, Shute and Ke's (2012) principles	Task learning/training performance, WM, inhibitory control, interference resolution, visuospatial reasoning, applied assessment, delay discounting, enjoyment, exerted effort
Nagle, Novak, et al. (2015)	Published article; Switzerland	Memory	Between-subject (pre-post) design; 2 groups (DDA, DDA-visual)	Older adults of assisted-living facilities	14	82.7	93	Assisted living facility	Tablet	1 week; none	3; 24 min	The Serious Game	DDA, feedback loops, story/theme	Flow theory	Game performance, number of rounds played, enjoyment, noticing the changes, liked the changes
Nagle, Riener, et al. (2015)	Published article; Switzerland	WM	Between-subject (pre-post) design; 2 groups (AUTO, USER-CONTROL)	Healthy older adults	21	69.9	48	Home	Tablet	3 weeks; none	Up to the participant; up to the participant	WM Training Game	Difficulty adjustment (dynamic or manual), rewards, story/theme	SDT	In-game performance, fluid intelligence, WM, compliance (motivation, total duration of play, frequency of paly), subjective appraisal of individual game elements
Ninaus et al. (2015)*	Published article; Austria	WM	Between-subject (post) design; 2 groups (NOGAME, GAME)	University students	30	23.80	80	Online	Computer	25 min; none	1; 25 min	GAME	Challenge, DDA, feedback loops, game level, progress, story/theme	Flow theory	Task performance, maximum WM capacity, state of flow
Notebaert et al. (2015)	Published article; Australia	Attention (anxiety)	Between-subject (pre-post) design; 4 groups (dot-probe ABMT attend-happy, dot-probe ABMT attend-angry, PIM ABMT attend-happy, PIM ABMT attend-angry)	Adults with moderate trait anxiety	83	21.00	72	Laboratory	Computer	NS; none	1; NS	PIM	Feedback loops, rewards	NS	Attentional bias, emotional vulnerability
Olfers and Band (2017)	Published article; the Netherlands	Attention, cognitive flexibility, WM	Between-subject (pre-post) design; 3 groups (flexibility, attention, control/active)	Healthy young adults	72	23.00 [a]	56 [a]	Online	Computer or laptop	4 weeks; none	20 (minimum 15); 45 min	Luminosity	Avatar, challenge, DDA, rewards, story/theme	NS	General performance, attentional performance, visual short-term memory
Pieters et al. (2017)- Study 1	Published article; Belgium	Attention (mood)	Between-subject (pre-post) design; 2 groups (IMPACT attend positive, IMPACT attend negative)	Healthy undergraduate students	58	23.53	83	Laboratory	Computer	15 min; none	1; 15 min	IMPACT	Challenge, DDA, feedback loops, progress, rewards	NS	Attentional bias (engagement, disengagement), training performance, happy mood (stress reactivity), anxious mood (stress reactivity), sad mood (stress reactivity)
Pieters et al. (2017)- Study 2	Published article; Belgium	Attention (mood)	Between-subject (pre-post) design; 3 groups (IMPACT attend positive, IMPACT attend negative, no training)	Healthy undergraduate students	82	21.89	82	Laboratory	Computer	5 days; none	5; 15 min	IMPACT	Challenge, DDA, feedback loops, progress, rewards	NS	Attentional bias (engagement, disengagement), attentional control (alerting, orienting, executive control), training performance, happy mood (stress reactivity), anxious mood (stress reactivity), sad mood (stress reactivity), anhedonic depression symptoms, anxious arousal symptoms, general distress symptoms, brooding symptoms, reflection symptoms
Prins et al. (2011)*	Published article; the Netherlands	WM	Between-subject (pre-post) design; 2 groups (game, control)	Children with ADHD on the waiting list for ADHD treatment	51	9.47	18	Training room at 3 outpatient mental healthcare institutions	Laptop	3 weeks; none	3; minimum 15 min and maximum 35 min	Supermecha	Avatar, DDA, feedback loops, game level, rewards, story/theme	Gee's (2005) principles	Training performance, visuospatial WM, motivation
Sahakian et al. (2015)	Published article; United Kingdom	Episodic memory	Between-subject (pre-post) design; 2 groups (cognitive training, treatment as usual)	Adults with a diagnosis of schizophrenia	22	28.48	50	NS	iPad	4 weeks; none	NS; NS	Wizard	Avatar, DDA, feedback loops, game level, rewards, sound effects, story/theme	NS	Episodic memory, functioning in activities of daily living, motivation to continue playing the game, enjoyment
Savulich et al. (2017)	Published article; United Kingdom	Episodic memory	Between-subject (pre-post) design; 2 groups (Game Show, clinic visits as usual)	Patients with a diagnosis of amnestic mild cognitive impairment	42	76.05	40	NS	iPad	4 weeks; none	NS; 60 min	Game Show	Challenge, DDA, game level, rewards, sound effects	NS	Episodic memory, visuospatial memory, general cognition, neuropsychiatric symptoms, general alertness, self-confidence, self-reported memory ability, enjoyment, motivation
Scase et al. (2017)	Conference paper; United Kingdom	Attention, EFs, memory, praxis	Between-subject (post) design; 2 groups (retirement village, living separately)	Older adults with mild cognitive impairment	24	75.13	92	Home	Tablet	47 days; none	2-59; 29 min (mean)	Find it, match it, solve it, complete it	Progress, rewards, story/theme	Operant conditioning, Flow theory	Adherence, user experience

Author (year)	Publication type; country	Cognitive domain	Study design	Participant characteristics				Intervention characteristics				Gamification characteristics			Outcomes
				Type	*N*	M_{age}	% female	Study setting	Study device	Study duration; follow-up	*N* session; session length	Game name	Game element	Theory used	
Souders et al. (2017)	Published article; United States	Inductive reasoning, planning, spatial reasoning, speed processing, task-switching, WM	Between-subject (pre-post) design; 2 groups (Mind Frontiers, active control)	Older adults	60	72.35	57	Home	Tablet	1 month; none	30; 45 min	Mind Frontiers	Challenge, DDA, feedback loops, game level, progress, rewards, story/theme	Unified theory of acceptance and use of technology, technology acceptance model	Game performance, reasoning ability, processing speed, executive control, memory, adherence
Trapp et al. (2008)	Published article; Germany	Attention, problem solving, verbal memory, visuomotor, visuospatial memory	Between-subject (pre-post) design; 2 groups (experimental, control)	Schizophrenia patients	40	32.03	75	NS (under supervision)	Computer	10 weeks; none	20, NS	X-Cog	Avatar, DDA, feedback loops, game level, sound effects, story/theme	NS	Problem solving, sustained attention, selective attention, WM, verbal memory, positive symptoms, negative symptoms, depressive symptoms, paranoid symptoms, trait anxiety, state anxiety, enjoyment, helpful
Trapp et al. (2013)	Published article; Germany	Attention, problem solving, verbal memory, visuomotor, visuospatial memory	Between-subject (pre-post) design; 2 groups (experimental, control)	Schizophrenia patients	60	36.65	50	Hospital	Computer	3 weeks; 5 years	12; 60 min	X-Cog	Avatar, DDA, feedback loops, game level, sound effects, story/theme	NS	Attention, verbal memory, visual memory, problem solving, speed of processing, hallucinations, delusions, bizarre behaviour, positive formal thought disorder, affective flattening or blunting, alogia, avolition/apathy, anhedonia/asociality, paranoid thinking, depression
van der Oord et al. (2014)	Published article; Belgium	Cognitive flexibility, inhibition, WM	Between-subject (pre-post) design; 2 groups (active treatment, wait-list)	Children with ADHD	40	9.75	18	Home	Computer	5 weeks; 9 weeks	25; about 40 min	Braingame Brian	Avatar, challenge, DDA, feedback loops, game level, progress, rewards, sound effects, story/theme	NS	ADHD symptoms, EFs (inhibition, cognitive flexibility, WM, metacognition)
Verbeken et al. (2013)	Published article; Belgium	Inhibition, WM	Between-subject (pre-post) design; 2 groups (EF-training, care as usual)	Inpatient obese children	43	9.79 [a]	50 [a]	Clinic	Computer	6 weeks; 8 and 12 weeks	25; 40 min	Braingame Brian	Avatar, challenge, DDA, feedback loops, game level, progress, rewards, sound effects, story/theme	NS	EFs (inhibition, WM, metacognition), visuospatial WM, BMI, acceptability of the training, enjoyment, motivation
Vourvopoulos et al. (2014)	Conference paper; Portugal	Attention, EFs, visuospatial orientation	Single group design	Patients with cognitive deficits	10	NS	80	Hospital	Computer with joystick	NS; none	10; 20 min	RehabCity	Challenge, DDA, feedback loops, game level, rewards, story/theme	NS	Usability (effectiveness, efficiency, satisfaction levels)

Note. NS= Not Specified; WM= Working Memory; DDA= Dynamic Difficulty Adjustment; EF= Executive Function; SDT= Self-determination Theory; GNG= Go/No-Go; VPT= Visual Probe Task; VST= Visual Search Task; ADHD= Attention Deficit Hyperactivity Disorder; ABMT= Attentional Bias Modification Training; IMPACT= Intrinsically-Motivating Playable Attentional Control Training; BCI= Brain-computer Interface; MT-PRO= Mindtastic Procrastination; PIM= Person-identity-matching.
*included in the meta-analysis
[a]before drop-out

Table 2.2. Number of game elements used in the selected studies.

Number of game elements used	Count (%)
1 element	0 (0%)
2 elements	3 (6%)
3 elements	7 (14%)
4 elements	4 (8%)
5 elements	14 (29%)
6 elements	7 (14%)
7 elements	6 (12%)
8 elements	3 (6%)
9 elements	5 (10%)

Table 2.3. Ratings of methodological quality for each study included in the systematic review.

	External Validity[a]			Internal Validity[a]			
Author (year)	Gamified task	Setting and/or location of the study	Total	Blinding of participants	Blinding of personnel	Control comparison condition	Total
Abellanoza (2017)	1	1	2	Unclear (0)	Unclear (0)	0	0
Baniqued et al. (2015)	1	1	2	0	Unclear (0)	0	0
Birk et al. (2016)	1	1	2	Unclear (0)	Unclear (0)	0	0
Boendermaker et al. (2015)- Study 1*	1	1	2	0	0	1	1
Boendermaker et al. (2016)*	1	1	2	0	0	1	1
Boendermaker et al. (2017)*	1	1	2	0	0	1	1
Boletsis and McCallum (2016)	1	1	2	Unclear (0)	0	0	0
Boot et al. (2016)	1	0	1	Unclear (0)	Unclear (0)	0	0
Brezovszky et al. (2013)	1	0	1	Unclear (0)	0	0	0
Choi and Medalia (2010)*	1	1	2	0	1	1	2
Connor and Shaw (2016)	1	1	2	Unclear (0)	0	0	0
Dassen et al. (2017)	1	1	2	Unclear (0)	Unclear (0)	0	0
De Vries et al. (2015)	1	1	2	1	1	0	2
Dennis and O'Toole (2014)	1	1	2	1	1	0	2
Dennis-Tiwary et al. (2017)	1	1	2	1	1	0	2
Dennis-Tiwary et al. (2016)	1	1	2	1	1	0	2
Dorrenbacher et al. (2014)*	1	1	2	1	0	1	2
Double and Birney (2016)	0	1	1	0	0	0	0
Dovis et al. (2015)	1	1	2	1	1	0	2
Enock (2015)	1	1	2	Unclear (0)	Unclear (0)	0	0
Garolera et al. (2015)	0	0	0	Unclear (0)	Unclear (0)	0	0
Gehring et al. (2011)	1	1	2	0	0	0	0
Gehring et al. (2009)	1	1	2	0	0	0	0
Hiraoka et al. (2016)	0	1	1	Unclear (0)	0	0	0
Jaeggi et al. (2011)	1	0	1	Unclear (0)	Unclear (0)	0	0
Katz et al. (2014)*	1	1	2	Unclear (0)	Unclear (0)	1	1
Kiili et al. (2013)- Study 1	1	0	1	Unclear (0)	0	0	0
Kiili et al. (2013)- Study 2	1	1	2	Unclear (0)	0	0	0
Kühn et al. (2017)	1	1	2	Unclear (0)	Unclear (0)	0	0
Lee et al. (2013)	1	1	2	0	0	0	0
Lukas and Berking (2017)	0	1	1	0	0	0	0
Mohammed et al. (2017)*	1	1	2	1	1	1	3
Nagle, Novak, et al. (2015)	1	1	2	Unclear (0)	Unclear (0)	0	0
Nagle, Riener, et al. (2015)	1	1	2	Unclear (0)	Unclear (0)	0	0
Ninaus et al. (2015)*	1	0	1	1	0	1	2
Notebaert et al. (2015)	1	1	2	Unclear (0)	Unclear (0)	0	0
Olfers and Band (2017)	1	1	2	1	Unclear (0)	0	1
Pieters et al. (2017)- Study 1	1	1	2	Unclear (0)	Unclear (0)	0	0
Pieters et al. (2017)- Study 2	1	1	2	Unclear (0)	Unclear (0)	0	0
Prins et al. (2011)*	1	1	2	Unclear (0)	0	1	1
Sahakian et al. (2015)	0	0	0	1	Unclear (0)	0	1
Savulich et al. (2017)	1	0	1	Unclear (0)	Unclear (0)	0	0
Scase et al. (2017)	1	1	2	Unclear (0)	Unclear (0)	0	0
Souders et al. (2017)	1	1	2	Unclear (0)	Unclear (0)	0	0
Trapp et al. (2008)	0	0	0	Unclear (0)	0	0	0
Trapp et al. (2013)	1	1	2	1	1	0	2
van der Oord et al. (2014)	1	1	2	Unclear (0)	1	0	1
Verbeken et al. (2013)	1	1	2	Unclear (0)	1	0	1
Vourvopoulos et al. (2014)	1	1	2	0	0	0	0

[a] Range: 0= at least one of the applicable criteria is not fulfilled; 1= all applicable criteria are fulfilled.
* Included in the meta-analysis

References

Records with an * have been included in the systematic review and with an ** in the meta-analysis.

*Abellanoza, C. K. (2017). *Rewind-remind: Investigating how gamification of memory tasks can evaluate associative memory performance in healthy, older adults.* (Doctoral dissertation). Available from Proquest Dissertations & Theses Global database. (Order No. 10629254)

*Baniqued, P. L., Allen, C. M., Kranz, M. B., Johnson, K., Sipolins, A., Dickens, C., . . . Kramer, A. F. (2015). Working memory, reasoning, and task switching training: Transfer effects, limitations, and great expectations? *PLoS ONE, 10*(11), e0142169. doi:10.1371/journal.pone.0142169

*Birk, M. V., Mandryk, R. L., & Atkins, C. (2016, October). *The motivational push of games: The interplay of intrinsic motivation and external rewards in games for training.* Paper presented at the Proceedings of the Annual Symposium on Computer-Human Interaction in Play (CHI PLAY), Texas, USA.

**Boendermaker, W. J., Boffo, M., & Wiers, R. W. (2015). Exploring elements of fun to motivate youth to do cognitive bias modification. *Games for Health, 4*(6), 434-443. doi:10.1089/g4h.2015.0053

**Boendermaker, W. J., Sanchez Maceiras, S., Boffo, M., & Wiers, R. W. (2016). Attentional bias modification with serious game elements: Evaluating the shots game. *JMIR Serious Games, 4*(2), e20. doi:10.2196/games.6464

**Boendermaker, W. J., Veltkamp, R. C., & Peeters, M. (2017). Training behavioral control in adolescents using a serious game. *Games for Health Journal, 6*(6), 351-357. doi:10.1089/g4h.2017.0071

*Boletsis, C., & McCallum, S. (2016). Augmented reality cubes for cognitive gaming: Preliminary usability and game experience testing. *International Journal of Serious Games, 3*(1), 3-18. doi:10.17083/ijsg.v3i1.106

*Boot, W. R., Souders, D., Charness, N., Blocker, K., Roque, N., & Vitale, T. (2016). The gamification of cognitive training: Older adults' perceptions of and attitudes toward digital game-based interventions. In J. Zhou & G. Salvendy (Eds.), Human Aspects of IT for the Aged Population. Design for Aging. ITAP 2016. (Vol. 9754, pp. 290-300). Toronto, Canada: Springer, Cham. doi:10.1007/978-3-319-39943-0_28

*Brezovszky, B., Lehtinen, E., McMullen, J., Rodriguez, G., & Veermans, K. (2013, October). *Training flexible and adaptive arithmetic problem solving skills through exploration with numbers: The development of numbernavigation game.* Paper presented at the Proceedings of the 7th European Conference on Games Based Learning (ECGBL), Porto, Portugal.

**Choi, J., & Medalia, A. (2010). Intrinsic motivation and learning in a schizophrenia spectrum sample. *Schizophr Res, 118*(1-3), 12-19. doi:10.1016/j.schres.2009.08.001

*Connor, B. B. P., & Shaw, C. A. M. A. (2016). Case study series using brain-training games to treat attention and memory following brain injury. *Journal of Pain Management, 9*(3), 217-226. Retrieved from http://www.icdvrat.org/2014/papers/ICDVRAT2014_S08N3_Connor_etal.pdf

Cordova, D. I., & Lepper, M. R. (1996). Intrinsic motivation and the process of learning: Beneficial effects of contextualization, personalization, and choice. *Journal of Educational Psychology, 88*(4), 715-730. doi:10.1037/0022-0663.88.4.715

*Dassen, F. C. M., Houben, K., Van Breukelen, G. J. P., & Jansen, A. (2017). Gamified working memory training in overweight individuals reduces food intake but not body weight. *Appetite, 124*, 89-98. doi:10.1016/j.appet.2017.05.009

*De Vries, M., Prins, P. J. M., Schmand, B. A., & Geurts, H. M. (2015). Working memory and cognitive flexibility-training for children with an autism spectrum disorder: A randomized controlled trial. *Journal of Child Psychology and Psychiatry and Allied Disciplines, 56*(5), 566-576. doi:10.1111/jcpp.12324

*Dennis-Tiwary, T. A., Denefrio, S., & Gelber, S. (2017). Salutary effects of an attention bias modification mobile application on biobehavioral measures of stress and anxiety during pregnancy. *Biological Psychology, 127*, 148-156. doi:10.1016/j.biopsycho.2017.05.003

*Dennis-Tiwary, T. A., Egan, L. J., Babkirk, S., & Denefrio, S. (2016). For whom the bell tolls: Neurocognitive individual differences in the acute stress-reduction effects of an attention bias modification game for anxiety. *Behaviour Research and Therapy, 77*, 105-117. doi:10.1016/j.brat.2015.12.008

*Dennis, T. A., & O'Toole, L. J. (2014). Mental health on the go: Effects of a gamified attention-bias modification mobile application in trait-anxious adults. *Clinical Psychological Science, 2*(5), 576-590. doi:10.1177/2167702614522228

**Dorrenbacher, S., Muller, P. M., Troger, J., & Kray, J. (2014). Dissociable effects of game elements on motivation and cognition in a task-switching training in middle childhood. *Frontiers in Psychology, 5*(1275). doi:10.3389/fpsyg.2014.01275

*Double, K. S., & Birney, D. P. (2016). The effects of personality and metacognitive beliefs on cognitive training adherence and performance. *Personality and Individual Differences, 102*, 7-12. doi:10.1016/j.paid.2016.04.101

*Dovis, S., Van der Oord, S., Wiers, R. W., & Prins, P. J. (2015). Improving executive functioning in children with ADHD: training multiple executive functions within the context of a computer game. a randomized double-blind placebo controlled trial. *PLoS ONE, 10*(4), e0121651. doi:10.1371/journal.pone.0121651

*Enock, P. (2015). *Making an IMPACT: Designing and testing a novel attentional training game to reduce social anxiety.* (Doctoral dissertation). Available from Proquest Dissertations & Theses Global database. (Order No. 3738754)

*Garolera, M., Berga, N., Quintana, M., Chico, G., Cerulla, N., López, M., . . . Rimbau, J. (2015, November). *Active-U: Playing to stimulate your brain.* Paper presented at the 2nd International Workshop on Gamification in Health, Barcelona, Spain.

Gee, J. P. (2005). Learning by design: Good video games as learning machines. *E-Learning and Digital Media, 2*(1), 5-16. doi:10.2304/elea.2005.2.1.5

*Gehring, K., Aaronson, N., Taphoorn, M., & Sitskoorn, M. (2011). A description of a cognitive rehabilitation programme evaluated in brain tumour patients with mild to moderate cognitive deficits. *Clinical Rehabilitation, 25*(8), 675-692. doi:10.1177/0269215510395791

*Gehring, K., Sitskoorn, M. M., Gundy, C. M., Sikkes, S. A. M., Klein, M., Postma, T. J., . . . Aaronson, N. K. (2009). Cognitive rehabilitation in patients with gliomas: a randomized, controlled trial. *Journal of Clinical Oncology: Official Journal of the American Society of Clinical Oncology, 27*(22), 3712-3722. doi:10.1200/jco.2008.20.5765

*Hiraoka, T., Wang, T. W., & Kawakami, H. (2016, August, September). *Cognitive function training system using game-based design for elderly drivers.* Paper presented at the 13th International Federation of Automatic Control Symposium on Analysis, Design, and Evaluation of Human-Machine Systems, Kyoto, Japan.

*Jaeggi, S. M., Buschkuehl, M., Jonides, J., & Shah, P. (2011). Short- and long-term benefits of cognitive training. *Proceedings of the National Academy of Sciences of the United States of America, 108*(25), 10081-10086. doi:10.1073/pnas.1103228108

**Katz, B., Jaeggi, S., Buschkuehl, M., Stegman, A., & Shah, P. (2014). Differential effect of motivational features on training improvements in school-based cognitive training. *Frontiers in Human Neuroscience, 8*(242). doi:10.3389/fnhum.2014.00242

*Kiili, K., Ninaus, M., Koskela, M., Tuomi, M., & Lindstedt, A. (2013, January). *Developing games for health impact: Case brains vs zombies.* Paper presented at the Proceedings of the 7th European Conference on Games Based Learning (ECGBL), Porto, Portugal.

*Kiili, K., & Perttula, A. (2013). A design framework for educational exergames. In S. d. Freitas, M. Ott, M. M. Popescu, & I. Stanescu (Eds.), *New Pedagogical Approaches in Game Enhanced Learning: Curriculum Integration* (pp. 136-158). Hershey, PA, USA: IGI Global.

*Kühn, S., Lorenz, R. C., Weichenberger, M., Becker, M., Haesner, M., O'Sullivan, J., . . . Gallinat, J. (2017). Taking control! Structural and behavioural plasticity in response to game-based inhibition training in older adults. *NeuroImage, 156*, 199-206. doi:10.1016/j.neuroimage.2017.05.026

*Lee, T.-S., Goh, S. J. A., Quek, S. Y., Phillips, R., Guan, C., Cheung, Y. B., . . . Krishnan, K. R. R. (2013). A brain-computer interface based cognitive training system for healthy elderly: A randomized control pilot study for usability and preliminary efficacy. *PLoS ONE, 8*(11), e79419. doi:10.1371/journal.pone.0079419

*Lukas, C. A., & Berking, M. (2017). Reducing procrastination using a smartphone-based treatment program: A randomized controlled pilot study. *Internet Interventions, 12*, 83-90. doi:10.1016/j.invent.2017.07.002

**Mohammed, S., Flores, L., Deveau, J., Cohen Hoffing, R., Phung, C., M. Parlett, C., . . . R. Seitz, A. (2017). The benefits and challenges of implementing motivational features to boost cognitive training outcome. *Journal of Cognitive Enhancement, 1*(4), 491-507. doi:10.1007/s41465-017-0047-y

*Nagle, A., Novak, D., Wolf, P., & Riener, R. (2015). Increased enjoyment using a tablet-based serious game with regularly changing visual elements: A pilot study. *Gerontechnology, 14*(1), 32-44. doi:10.4017/gt.2015.14.1.001.00

*Nagle, A., Riener, R., & Wolf, P. (2015). High user control in game design elements increases compliance and in-game performance in a memory training game. *Frontiers in Psychology, 6*, 1-15. doi:10.3389/fpsyg.2015.01774

**Ninaus, M., Pereira, G., Stefitz, R., Prada, R., Paiva, A., Neuper, C., & Wood, G. (2015). Game elements improve performance in a working memory training task. *International Journal of Serious Games, 2*(1), 3-16. doi:10.17083/ijsg.v2i1.60

*Notebaert, L., Clarke, P. J. F., Grafton, B., & MacLeod, C. (2015). Validation of a novel attentional bias modification task: The future may be in the cards. *Behaviour Research and Therapy, 65*, 93-100. doi:10.1016/j.brat.2014.12.007

*Olfers, K. J. F., & Band, G. P. H. (2017). Game-based training of flexibility and attention improves task-switch performance: near and far transfer of cognitive training in an EEG study. *Psychological Research, 82*(1), 186-202. doi:10.1007/s00426-017-0933-z

*Pieters, E. K., De Raedt, R., Enock, P. M., De Putter, L. M. S., Braham, H., McNally, R. J., & Koster, E. H. W. (2017). Examining a novel gamified approach to attentional retraining: Effects of single and multiple session training. *Cognitive Therapy and Research, 41*(1), 89-105. doi:10.1007/s10608-016-9803-z

**Prins, P. J. M., Dovis, S., Ponsioen, A., ten Brink, E., & van der Oord, S. (2011). Does computerized working memory training with game elements enhance motivation and training efficacy in children with ADHD? *Cyberpsychology, Behavior, and Social Networking, 14*(3), 115-122. doi:10.1089/cyber.2009.0206

*Sahakian, B. J., Bruhl, A. B., Cook, J., Killikelly, C., Savulich, G., Piercy, T., . . . Jones, P. B. (2015). The impact of neuroscience on society: cognitive enhancement in

neuropsychiatric disorders and in healthy people. *Philosophical Transactions of the Royal Society B: Biological Sciences, 370*(1677), 20140214. doi:10.1098/rstb.2014.0214

*Savulich, G., Piercy, T., Fox, C., Suckling, J., Rowe, J. B., O'Brien, J. T., & Sahakian, B. J. (2017). Cognitive training using a novel memory game on an ipad in patients with amnestic mild cognitive impairment (aMCI). *International Journal of Neuropsychopharmacology, 20*(8), 624-633. doi:10.1093/ijnp/pyx040

*Scase, M., Marandure, B., Hancox, J., Kreiner, K., Hanke, S., & Kropf, J. (2017). Development of and adherence to a computer-based gamified environment designed to promote health and wellbeing in older people with mild cognitive impairment. In D. Hayn & G. Schreier (Eds.), *Health informatics meets eHealth: Digital insight - information-driven health & care. Proceedings of the 11th eHealth 2017 conference* (Vol. 236, pp. 348-355). Amsterdam, Netherlands: IOS Press BV.

Shute, V. J., & Ke, F. (2012). Games, Learning, and Assessment. In D. Ifenthaler, D. Eseryel, & X. Ge (Eds.), *Assessment in Game-Based Learning: Foundations, Innovations, and Perspectives* (pp. 43-58). New York, NY: Springer New York.

*Souders, D. J., Boot, W. R., Blocker, K., Vitale, T., Roque, N. A., & Charness, N. (2017). Evidence for narrow transfer after short-term cognitive training in older adults. *Frontiers in Aging Neuroscience, 9*(41), 1-10. doi:10.3389/fnagi.2017.00041

*Trapp, W., Hasmann, A., Gallhofer, B., Schwerdtner, J., Guenther, W., & Dobmeier, M. (2008). Cognitive improvement of schizophrenia patients: Enhancing cognition while enjoying computer-aided cognitive training. *Clinical Schizophrenia & Related Psychoses, 1*(4), 307-316. doi:10.3371/CSRP.1.4.2

*Trapp, W., Landgrebe, M., Hoesl, K., Lautenbacher, S., Gallhofer, B., Günther, W., & Hajak, G. (2013). Cognitive remediation improves cognition and good cognitive

performance increases time to relapse – results of a 5 year catamnestic study in schizophrenia patients. *BMC Psychiatry, 13*(1), 184-193. doi:10.1186/1471-244x-13-184

*van der Oord, S., Ponsioen, A. J., Geurts, H. M., Ten Brink, E. L., & Prins, P. J. (2014). A pilot study of the efficacy of a computerized executive functioning remediation training with game elements for children with ADHD in an outpatient setting: outcome on parent- and teacher-rated executive functioning and ADHD behavior. *Journal of Attention Disorders, 18*(8), 699-712. doi:10.1177/1087054712453167

*Verbeken, S., Braet, C., Goossens, L., & van der Oord, S. (2013). Executive function training with game elements for obese children: A novel treatment to enhance self-regulatory abilities for weight-control. *Behaviour Research and Therapy, 51*(6), 290-299. doi:10.1016/j.brat.2013.02.006

*Vourvopoulos, A., Faria, A. L., Ponnam, K., & Bermudez I Badia, S. (2014, November). *RehabCity: Design and validation of a cognitive assessment and rehabilitation tool through gamified simulations of activities of daily living.* Paper presented at the 11th Advances in Computer Entertainment Technology Conference, Madeira, Portugal.
