# Supplementary material for: The Effects of Gamification on Computerized Cognitive Training: Systematic Review and Meta-Analysis
Source: JMIR Serious Games. 2020 Aug 10;8(3):e18644. doi: 10.2196/18644 (PMC7445616; doi:10.2196/18644)

Multimedia Appendix 3: Meta-Analysis Tables and Figures

1. Meta-Analysis Tables

Table 3.1. Sensitivity analysis for motivation/engagement-related outcomes, excluding one study at a time.

Model	Study name	Statistics with study removed				
		Hedges g	Standard error	Lower limit	Upper limit	*P* value
	Boendermaker et al. (2015)- Study 1	0.82	0.25	0.32	1.32	.001
	Boendermaker et al. (2016)	0.83	0.25	0.34	1.32	.001
	Boendermaker et al. (2017)	0.66	0.26	0.14	1.18	.013
	Choi and Medalia (2010)	0.59	0.23	0.14	1.05	.011
	Dorrenbacher et al. (2014)	0.69	0.26	0.18	1.20	.008
	Katz et al. (2014)	0.82	0.25	0.33	1.31	.001
	Mohammed et al. (2017)	0.78	0.28	0.23	1.33	.005
	Prins et al. (2011)	0.60	0.24	0.14	1.06	.011
Random		0.72	0.24	0.26	1.19	.002

Table 3.2. Sensitivity analysis for demand/difficulty-related outcomes, excluding one study at a time.

Model	Study name	Statistics with study removed				
		Hedges g	Standard error	Lower limit	Upper limit	*P* value
	Boendermaker et al. (2015)- Study 1	-0.32	0.24	-0.78	0.15	.18
	Katz et al. (2014)	-0.63	0.25	-1.11	-0.14	.011
	Prins et al. (2011)	-0.58	0.31	-1.19	0.04	.067
Random		-0.52	0.19	-0.89	-0.14	.007

Table 3.3. Sensitivity analysis for cognitive process outcomes, excluding one study at a time.

Model	Study name	Statistics with study removed				
		Hedges g	Standard error	Lower limit	Upper limit	*P* value
	Boendermaker et al. (2015)- Study 1	0.34	0.19	-0.03	0.71	.068
	Boendermaker et al. (2016)	0.30	0.20	-0.10	0.70	.14
	Boendermaker et al. (2017)	0.26	0.21	-0.16	0.68	.23
	Choi and Medalia (2010)	0.13	0.12	-0.11	0.36	.29
	Dorrenbacher et al. (2014)	0.30	0.19	-0.08	0.68	.12
	Katz et al. (2014)	0.33	0.19	-0.03	0.70	.076
	Mohammed et al. (2017)	0.30	0.21	-0.11	0.72	.15
	Ninaus et al. (2015)	0.24	0.20	-0.14	0.63	.22
	Prins et al. (2011)	0.22	0.20	-0.16	0.61	.26
Random		0.27	0.18	-0.08	0.62	.14

Table 3.4. Sensitivity analysis for clinical outcomes, excluding one study at a time.

Model	Study name	Statistics with study removed				
		Hedges g	Standard error	Lower limit	Upper limit	*P* value
	Boendermaker et al. (2015)- Study 1	-0.01	0.13	-0.26	0.25	.97
	Boendermaker et al. (2016)	0.04	0.17	-0.29	0.37	.82
	Boendermaker et al. (2017)	0.22	0.16	-0.09	0.53	.16
	Choi and Medalia (2010)	0.10	0.18	-0.27	0.46	.61
Random		0.07	0.13	-0.19	0.32	.61

2. Meta-Analysis Figures

Figure 3.1. Funnel plot for motivation/engagement-related outcomes. Circles indicate studies from the present sample positioned by their respective estimated effect size and standard error.

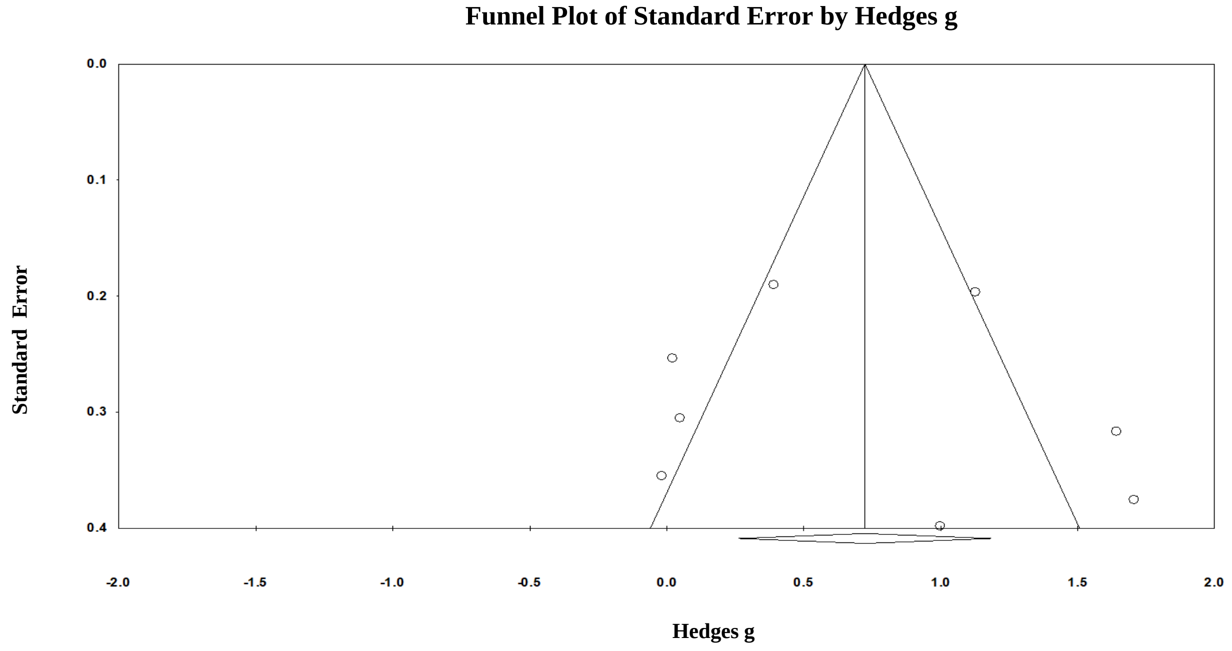

Figure 3.2. Funnel plot for demand/difficulty-related outcomes. Circles indicate studies from the present sample positioned by their respective estimated effect size and standard error.

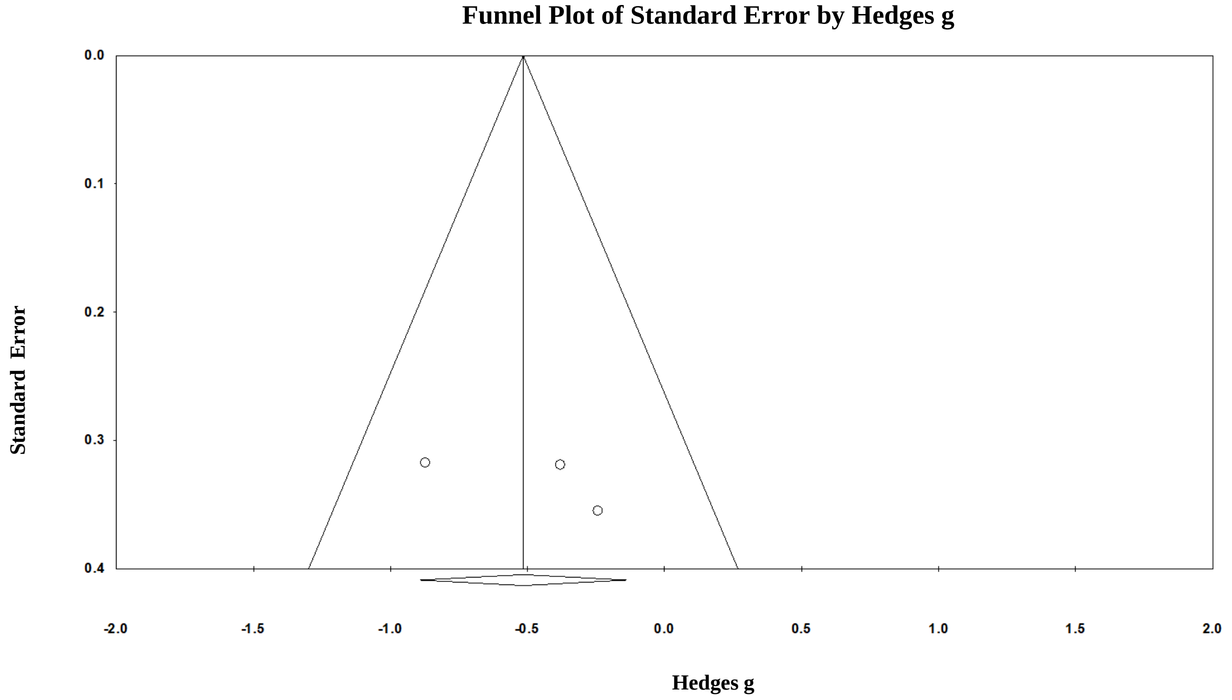

Figure 3.3. Funnel plot for cognitive process outcomes. Circles indicate studies from the present sample positioned by their respective estimated effect size and standard error.

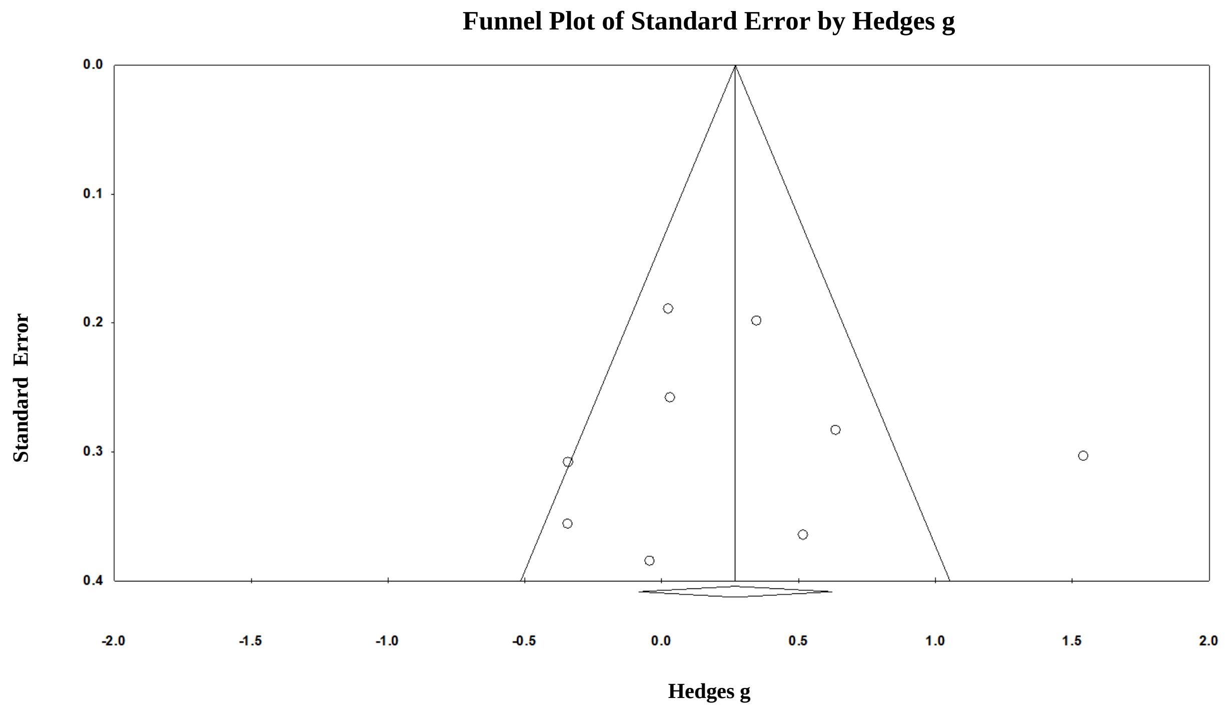

Figure 3.4. Funnel plot for clinical outcomes. Circles indicate studies from the present sample positioned by their respective estimated effect size and standard error.

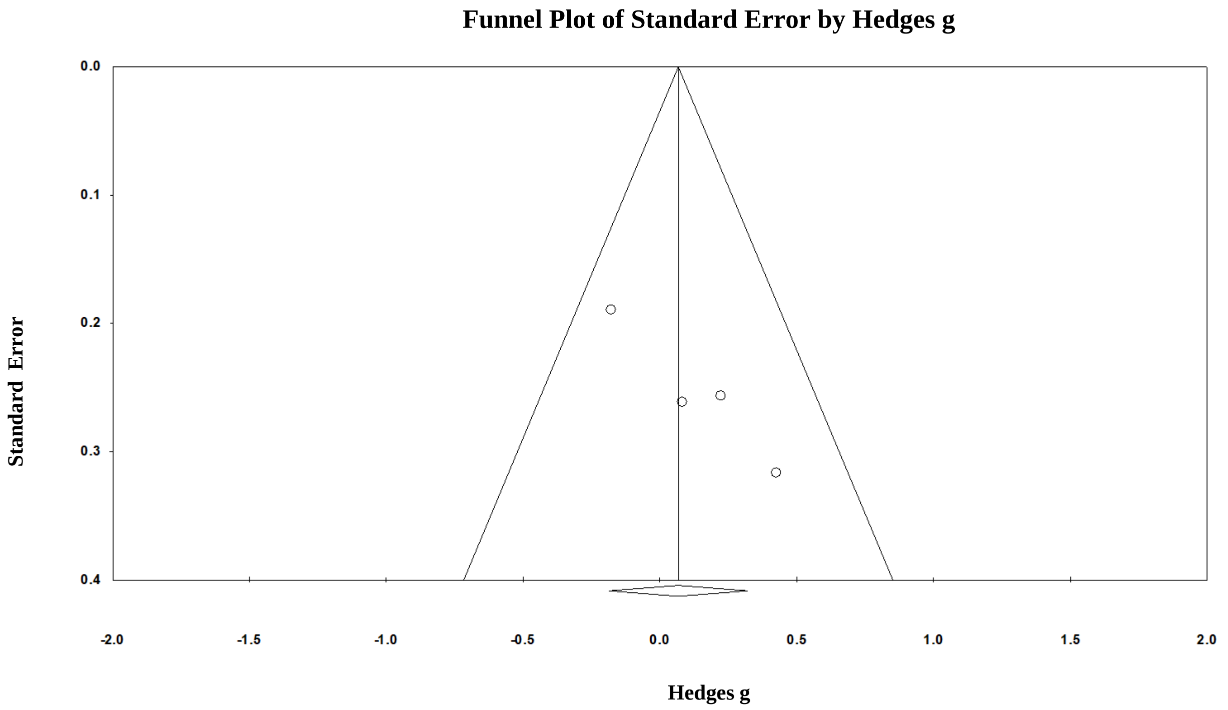

Figure 3.5. Meta-regression scatter plot of the relationship between the number of game elements used and effect sizes for motivation/engagement outcomes.

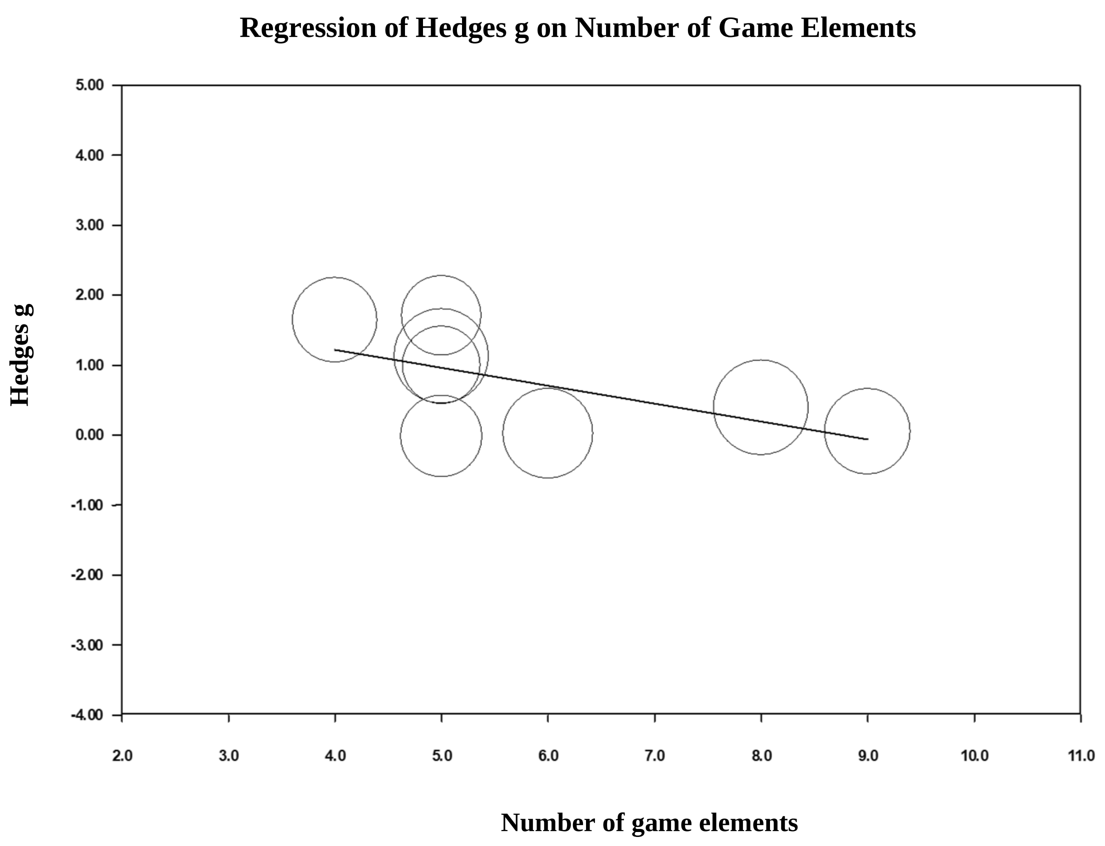

Figure 3.6. Meta-regression scatter plot of the relationship between the number of game elements used and effect sizes for cognitive process outcomes.

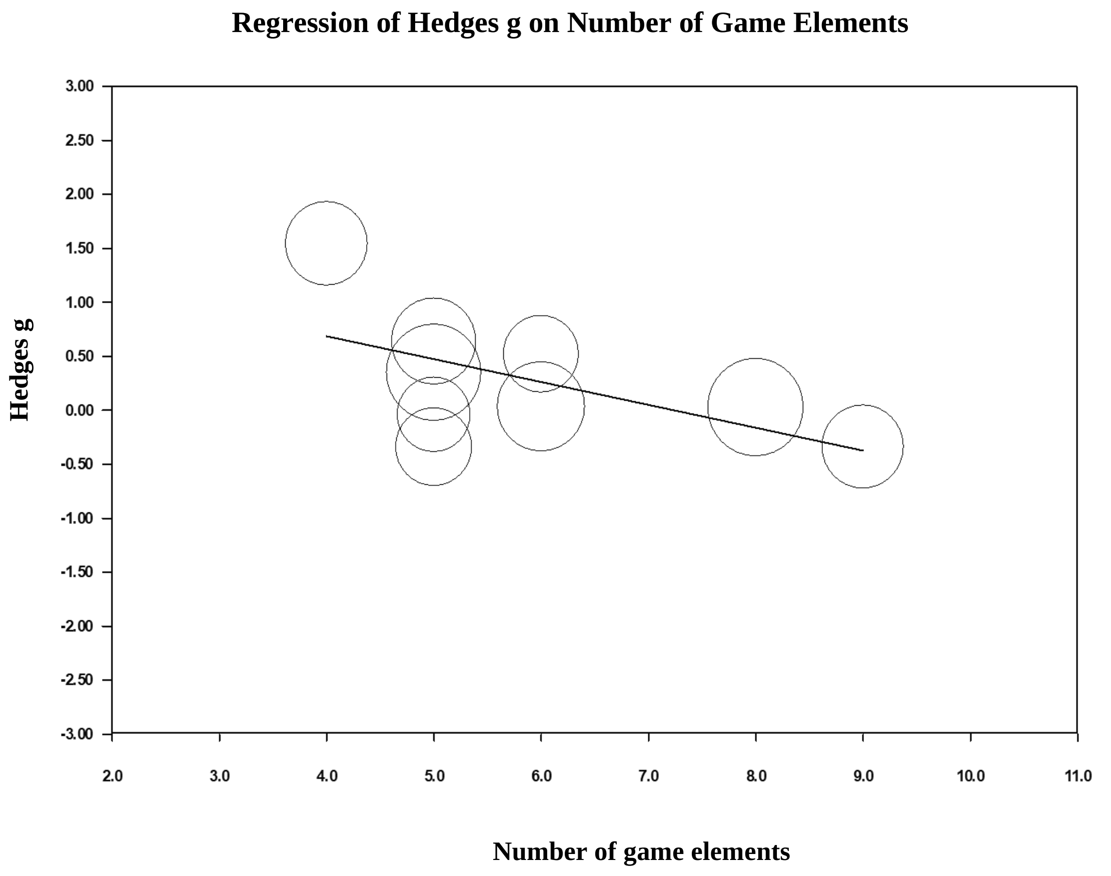

Figure 3.7. Meta-regression scatter plot of the relationship between the number of training sessions and effect sizes for motivation/engagement outcomes.

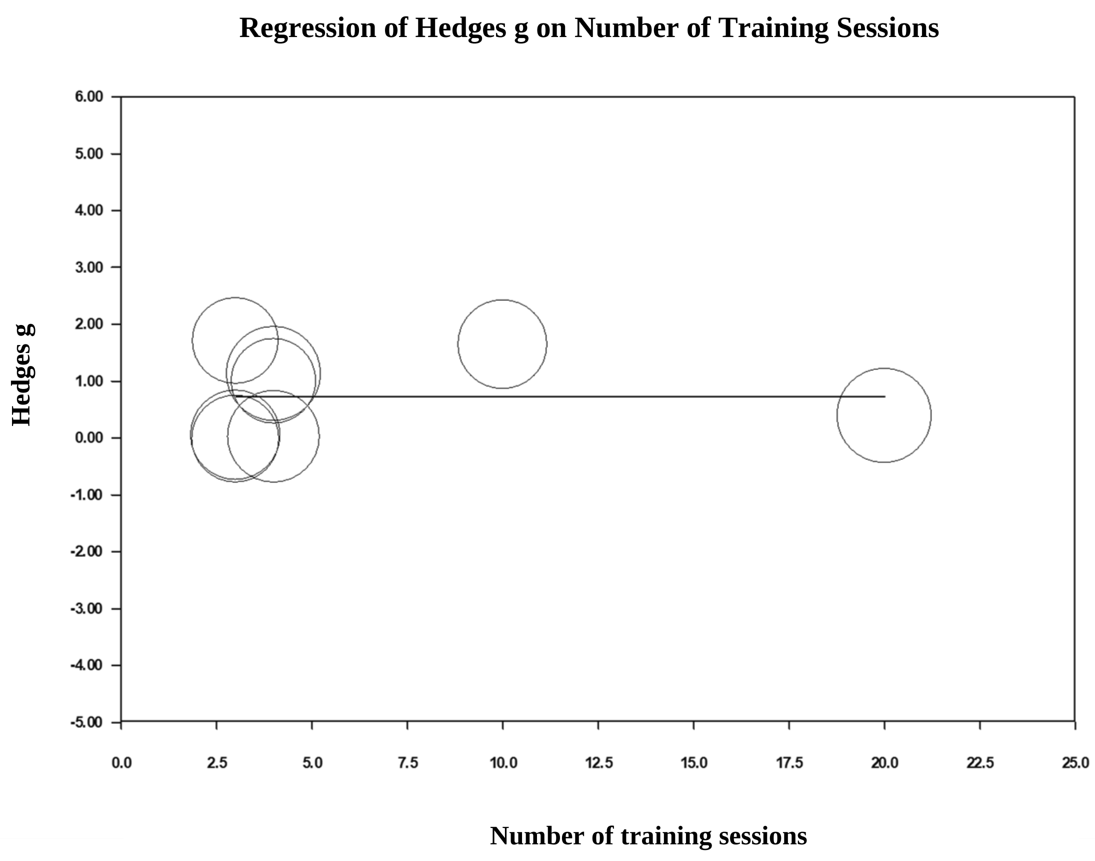

Figure 3.8. Meta-regression scatter plot of the relationship between the number of training sessions and effect sizes for cognitive process outcomes.

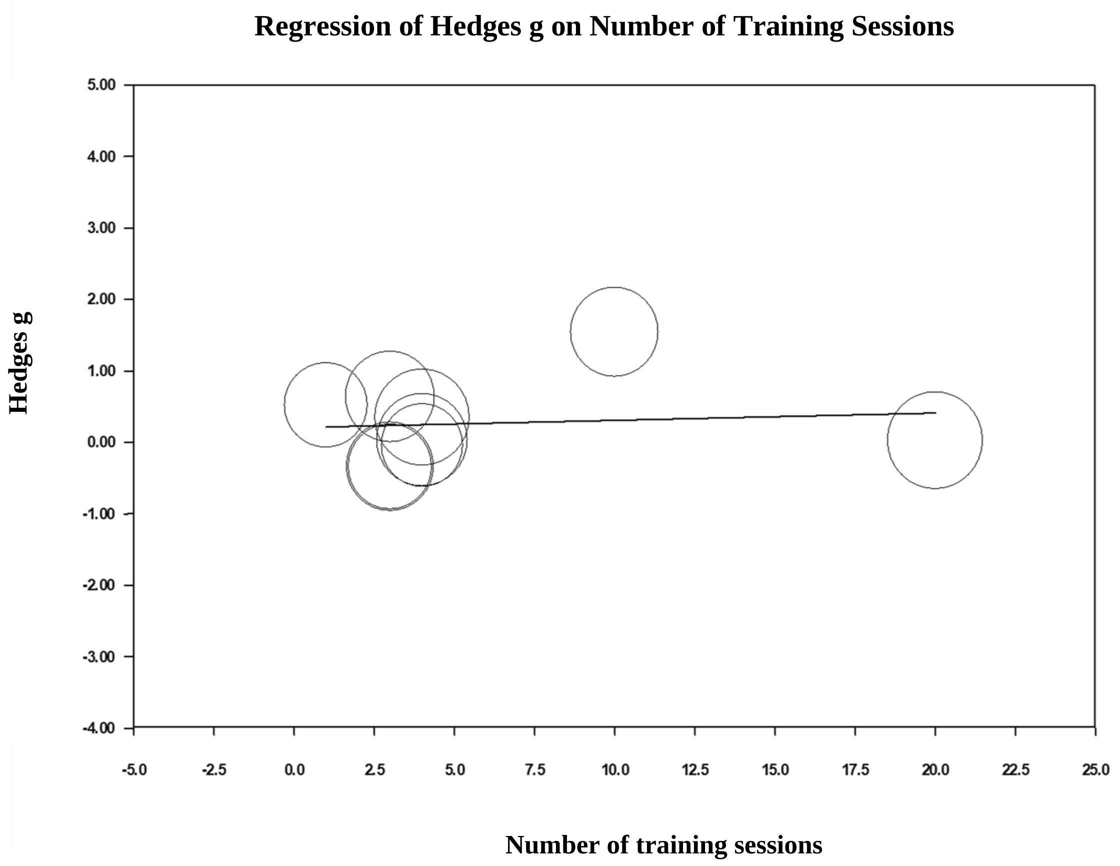

Supplement: Multimedia Appendix 3 [file games_v8i3e18644_app3.pdf]
